# Biodegradation of ^13^C‑Labeled and Nonlabeled Aliphatic–Aromatic Polyesters in Compost: Advancing Assessments through Combined Respirometry and Residual Polyester Analysis

**DOI:** 10.1021/acs.est.6c00032

**Published:** 2026-07-23

**Authors:** Flora Wille, Kevin Kleemann, Yao Zhou, Madalina Jaggi, Stefano M. Bernasconi, Hannah Kleyer, Davide Ciccarese, Johannes Zimmer, Kai O. Siegenthaler, Martin Worch, Glauco Battagliarin, Andreas Künkel, Michael Sander

**Affiliations:** † Institute of Biogeochemistry and Pollutant Dynamics, Department of Environmental Systems Sciences, ETH Zürich, 8092 Zürich, Switzerland; ‡ Geological Institute, Department of Earth and Planetary Sciences, ETH Zürich, 8092 Zürich, Switzerland; § Société des Produits Nestlé S.A., 1000 Lausanne, Switzerland; ∥ Biodegradation and Microplastics Research, 5184 BASF SE, 67056 Ludwigshafen am Rhein, Germany; ⊥ Eawag, Swiss Federal Institute of Aquatic Science and Technology, Überlandstrasse 133, 8600 Dübendorf, Switzerland

**Keywords:** compost, compostable plastics, biodegradability testing, residual polyesters, stable carbon isotope labeling, PBAT, PBSeT

## Abstract

Standard polymer biodegradability tests in industrial compost rely on running incubations at high initial polymer concentrations and exclusively determining polymer mineralization to carbon dioxide, while not analyzing the nonmineralized fraction of polymer-added carbon. This work presents an approach that combines the use of ^13^C-labeled polyesters in compost incubations to selectively track both mineralized and nonmineralized polyester ^13^C at low initial polyester concentrations with analysis of residual polyester during the incubations by compost solvent extraction coupled to proton nuclear magnetic resonance spectroscopy. Incubations with ^13^C-labeled poly­(butylene adipate-*co*-terephthalate) (PBAT) and poly­(butylene sebacate-*co*-terephthalate) (PBSeT) at low initial concentrations exhibited shorter initial lag-phases and higher relative rates of mineralization as compared to parallel standard incubations run at high initial concentrations of nonlabeled PBAT and PBSeT, suggesting that the latter used polyester amounts in excess of the metabolic capacity of the compost microbial community. Mass balance assessment on polyester-added ^13^C over the incubations confirmed accurate ^13^C tracking in both mineralized and nonmineralized pools, despite low initial polyester concentrations. Extraction of residual ^13^C-labeled and nonlabeled polyesters revealed substantial incorporation of polyester carbon into microbial biomass during compost incubations. Biodegradability tests that exclusively rely on mineralization measurements may therefore underestimate polymer biodegradation in compost.

## Graphical abstract

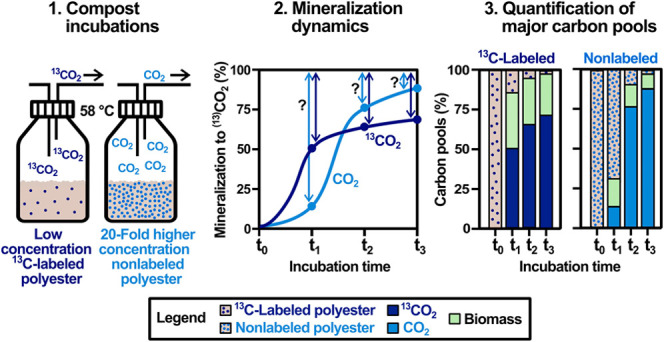

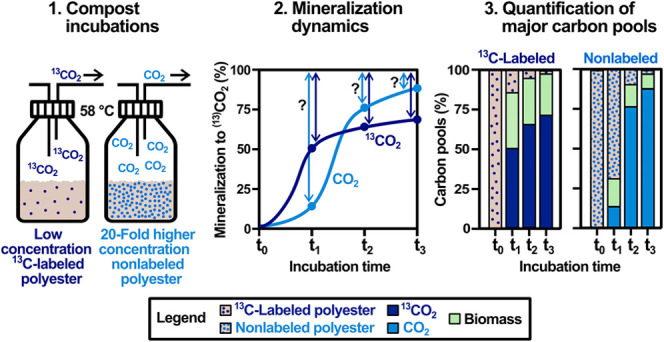

## Introduction

Biodegradable polymers are designed to undergo complete microbial metabolic utilization in targeted receiving environments, resulting in the full conversion of polymer carbon to carbon dioxide (CO_2_) and microbial biomass under aerobic conditions. Biodegradability is highly desired for polymer applications in which, after use, recollection and/or subsequent technical recycling or reuse of the polymer is impractical or unfeasible. Such cases include products intended for industrial composting systems (e.g., household biowaste bags, sticky labels on fruit and vegetables, coffee capsules, tea bags, and specific food-contact materials).^1–3^ For such applications, polymer biodegradability is critical to ensure that these polymers are effectively removed and that no persistent nano- and microplastic particles form during composting.

Many compostable plastic products contain the semicrystalline aliphatic–aromatic copolyester poly­(butylene adipate-*co*-terephthalate) (PBAT). A structurally related alternative is poly­(butylene sebacate-*co*-terephthalate) (PBSeT), which contains the biobased monomer sebacate instead of fossil-based adipate.^4–8^ Both polyesters have interesting material properties, including high elongation at break and impact resistance.^5,9^ PBAT biodegradability in compost,^10–13^ as well as other receiving environments,^14–17^ is well-documented, whereas studies on PBSeT biodegradability are scarcer and have mainly focused on soil and marine environments.^14,18,19^ The biodegradability of aliphatic–aromatic polyesters can be tuned by modifying their monomeric composition. For instance, soil biodegradability of PBAT decreases with an increasing molar ratio of terephthalic acid to aliphatic diacids, whereas replacing the adipate unit in PBAT with the longer diacid sebacate in PBSeT results in enhanced soil biodegradability.^14^

For being certified industrially compostable, polyesters must meet criteria specified in established certification standards, such as the EN 13432 (Europe),^20^ ASTM 6400 (United States),^21^ or ISO 17088 (international).^22^ A key criterion is aerobic biodegradability, defined as the conversion (i.e., mineralization) of at least 90% of polyester carbon to CO_2_ within six months of laboratory compost incubations under controlled conditions and constant temperature (typically 58 ± 2 °C). In these tests, biodegradation is assessed based on the extent of polyester mineralization to CO_2_, determined by respirometry.^23–25^ The 90% requirement reflects that a fraction of the polyester carbon can be incorporated into microbial biomass during biodegradation, such that complete biodegradation can be attained even though not all polyester carbon is converted to CO_2_.

However, exclusively relying on mineralization data can result in ambiguities in data interpretation.^26^ First, respirometric measurements are susceptible to artifacts resulting from so-called positive or negative ‘priming effects’,^27–30^ where added substrates (here: polyesters) stimulate or suppress the background mineralization of organic matter (here: compost), respectively. If positive or negative priming occurs, polyester-free control incubations no longer adequately capture background compost mineralization in incubations containing added polyesters, resulting in over- or underestimation of polyester mineralization, respectively. Priming may be particularly prominent in compost incubations given that they typically need to be set up at relatively high initial polyester concentrations to ensure accurate quantification of polyester-derived CO_2_ relative to high background compost organic matter (COM) mineralization. Second, mineralization data provide no information on the nature of the nonmineralized fraction of added polyester carbon. This fraction can include polyester-derived carbon that was incorporated into microbial biomass, in which case mineralization measurements underestimate the true extent of polyester biodegradation. Distinguishing biomass-incorporated carbon from residual polymer carbon is desirable, particularly when mineralization extents remain incomplete. Third, based on only respirometry, the mass balance on polymer-added carbon cannot be closed over the course of the incubation. Closed mass balances are, however, a central quality assurance criterion for accurately assessing biodegradation.

These ambiguities call for methodological extensions to the standard respirometric workflow used to assess polymer biodegradation in compost. A particularly promising workflow—recently introduced for soil and sediment incubations^4,31,32^ —combines the use of ^13^C-labeled polymers in incubations coupled to isotope-selective analytics, and solvent extraction coupled to quantitative proton nuclear magnetic resonance (^1^H NMR) spectroscopy to quantify residual polymer in the incubation matrix (an approach that itself does not require working with ^13^C-labeled polymers).^33,34^ The application of this approach to compost incubations would allow selective and sensitive quantification of mineralization of ^13^C-labeled polyesters to ^13^CO_2_ (using isotope-sensitive ^13^CO_2_ cavity ring down spectroscopy) even at low initial polyester concentrations, and, after termination of the incubations, quantification of the nonmineralized polyester-added ^13^C by elemental analysis of compost aliquots coupled to isotope ratio mass spectrometry (EA-IRMS). The sum of the nonmineralized and mineralized polyester-^13^C fractions then allows assessing whether the mass balance on polyester-added ^13^C is closed over the incubation. Conversely, the quantification of residual polyesters in compost by solvent extraction and ^1^H NMR enables assessing the relative contribution of residual polyester to the nonmineralized ^13^C fraction and, therefore, estimating the fraction of polyester carbon incorporated into microbial biomass. However, applying this workflow to compost incubations is expected to come with challenges due to the high COM content, which is expected to cause high background mineralization that can interfere with polyester-derived ^13^CO_2_ quantification, elevate CO_2_ background signals in EA-IRMS analysis of compost aliquots, and lead to coextraction of COM that interferes with residual polyester quantification by ^1^H NMR.

This work demonstrates the application of the extended workflow to the biodegradability assessment of PBAT and PBSeT in industrial compost based on conducting two sets of compost incubation experiments. The first set used custom-synthesized ^13^C-labeled PBAT and PBSeT as well as commercially available ^13^C-labeled cellulose (as positive biodegradation control) in compost incubations, all at low initial concentrations compared to concentrations used in standard tests. The second set used nonlabeled PBAT, PBSeT (with the same monomeric composition as the ^13^C-labeled analogues), and the polyester poly­(3-hydroxybutyrate-*co*-3-hydroxyhexanoate) (PHBH) (as positive control), all at high initial concentrations—closely matching the standard compost incubation setups used for biodegradability assessment. Each set included replicate incubations to quantify residual polyester (sets 1 and 2) and nonmineralized polyester-^13^C (set 1 only) at intermediate incubation times, allowing to estimate polyester carbon-derived biomass. The work demonstrates that the extended analytical workflow provides a comprehensive understanding of polyester biodegradation in industrial compost.

## Materials and Methods

### Chemicals

All chemicals were used as received. Details, including chemical purities and suppliers, are provided in the Supporting Information (SI), Section S1.

### Polymers

The ^13^C-labeled PBAT and PBSeT were synthesized at laboratory scale, as detailed in Section S2, Supporting Information, from a mixture of butanediol (B) (including both fully ^13^C_4_-labeled and nonlabeled B), terephthalic acid (T), and adipic acid (A) or sebacic acid (Se), respectively. Both ^13^C-labeled PBAT and PBSeT thus carried the ^13^C-label in the butanediol monomeric unit. The bulk polyester labeling degrees (%^13^C_substrate_) of ^13^C-labeled PBAT and PBSeT were approximately 12 and 11 atom % of the total polyester carbon atoms, respectively, as determined by EA-IRMS. The nonlabeled PBAT and PBSeT were from large-scale industrial syntheses. The T content (i.e., molar ratio of T to total diacid units (i.e., T and A or T and Se) were 46 and 47 mol % for ^13^C-labeled PBAT and PBSeT, respectively, and 47 and 48 mol % for nonlabeled PBAT and PBSeT, respectively, as determined by ^1^H NMR (Section S7, Supporting Information). Following synthesis, all polyesters were cryo-milled to powders and sieved to particle sizes between 100 and 300 μm for use in compost incubations. Size exclusion chromatography (SEC) analysis showed that the ^13^C-labeled PBAT and PBSeT had number-average molecular weights of *M*_n_ = 24.5 · 10^3^ g mol^–1^ and 19.0 · 10^3^ g mol^–1^, respectively, with dispersities (Đ) of 2.8 and 2.7. For the nonlabeled PBAT and PBSeT, *M*_n_ was 29.1 · 10^3^ and 29.1 · 10^3^ g mol^–1^ and Đ values were 3.7 and 3.9, respectively. Details on the SEC procedure and full molecular weight distributions are provided in Section S2, Supporting Information.

Powders of ^13^C-labeled cellulose (IsoLife, Netherlands; used as received; labeling extent: ∼97%) and nonlabeled PHBH (Kaneka Corporation, Belgium; used as received; 3-hydroxyhexanoate content of 9 mol %, as previously determined by ^1^H NMR^34^) were used as positive biodegradation controls for experimental sets 1 and 2, respectively.

All polymers and positive controls were stored in a desiccator at room temperature (∼21 °C), protected from light, until being used.

### Compost and Compost Inoculum

Batches of industrial compost (20 weeks aged, referred to as ‘very mature’ by vendor; sieved to 5 mm) and a high activity ‘young’ compost inoculum were purchased from Normec OWS, Belgium. Both were used within 2 days of delivery. Details on compost chemical characteristics and preparation for incubations and extractions are provided in Section S3, Supporting Information.

### Analytical Workflow to Assess Biodegradation

Set 1 of incubations was run with ^13^C-labeled PBAT, ^13^C-labeled PBSeT, and ^13^C-labeled cellulose, while set 2 used nonlabeled PBAT, PBSeT, and PHBH. Both sets had three groups of triplicate samples per substrate and triplicate compost blank samples. The analytical workflow for the two sets included (i) continuous quantification of mineralization of added substrates to ^(13)^CO_2_ during incubation, (ii) quantification of nonmineralized polyester- and cellulose-added ^13^C remaining in compost (set 1 only), and (iii) quantification of residual polyesters remaining in compost (sets 1 and 2). For (ii) and (iii), triplicate incubation bottles were terminated and analyzed after t_1_ = 4 days, t_2_ = 7 days, and t_3_ = 18 days of incubation (set 1) and after reaching mineralization extents of approximately 25%, 50%, and 90% (set 2) (which corresponded to t_1_ = 9, t_2_ = 14, and t_3_ = 23 days for PBAT; t_1_ = 9, t_2_ = 12, and t_3_ = 20 days for PBSeT; and t_1_ = 5, t_2_ = 8, and t_3_ = 23 days for PHBH). A schematic overview of the experimental setup and analytical workflow is provided in Section S4, Supporting Information.

#### Preparation of Incubation Bottles

For each sample, 120 g compost, ∼1.3 g compost inoculum, and, for bottles containing polyesters or cellulose, 351 ± 1 mg ^13^C-labeled polyesters or cellulose (set 1) or 8.0 ± 0.1 g nonlabeled polyesters (set 2) were thoroughly mixed in a stainless-steel bowl. Each mixed sample was then transferred into a 1 L glass incubation bottle. A total of 36 bottles were prepared per set (i.e., three groups (t_1_ to t_3_) of triplicates for each polyester/cellulose and blank compost). Samples were placed into the incubator within 60 min of preparation.

#### Incubation System and Quantification of Polyester and Cellulose Mineralization Extents, ^(13)^C_mineralized_

Mineralization of all samples was determined in a temperature-controlled (58 °C) incubation system (ECHO; Respirometer ER Series, ECHO Instruments, Slovenia). The incubation system contained 36 incubation lines connected to 36 incubation bottles. Incubations were run in continuous flow-through mode and the CO_2_ and ^13^CO_2_ in the bottle effluent gas were detected by an IR sensor of the ECHO system and, placed in series, a cavity ring down spectrometer (CRDS; G2201-i Isotopic Analyzer, Picarro, United States), respectively. A schematic depiction of the setup and system operation parameters are given in Section S5, Supporting Information. Each incubation line was analyzed approximately every 8 h for 15 min intervals, of which the ^(13)^CO_2_ data for the last 5 min were time-averaged and used for the calculation of mineralization rates. The obtained mineralization rates were integrated over time to obtain mineralization extents, ^(13)^C_mineralized_. All calculations were conducted as previously published^14,31^ and are presented in Section S5, Supporting Information.

#### Compost Subsampling for Analysis of Nonmineralized Residual Substrate Carbon

Triplicate incubation bottles (compost with added polyester, cellulose, and blank compost controls) from each set were removed from the incubators at time points t_1_, t_2_, and t_3_ (see above), frozen at −20 °C, and subsequently freeze-dried (0.1 mbar for at least 48 h). The freeze-dried compost in each bottle was homogenized by gentle stirring with a spatula, then transferred onto glassine weighing paper without further stirring to prevent demixing, and immediately divided into four approximately equally sized quarters.

For quantification of nonmineralized polyester- and cellulose-added ^13^C in compost (^13^C_nonmineralized_), a ∼9 g aliquot was taken from the first quarter for further homogenization and subsampling for EA-IRMS analysis (see section below). For quantification of residual polyester (^(13)^C_residual polyester_), two separate ∼9 g aliquots (set 1) or one ∼15–20 g aliquot (set 2)—both corresponding to ∼25 wt % of the total compost sample—were taken from the second quarter. Each aliquot was transferred into a cellulose thimble for Soxhlet extraction (see section below). The remaining compost was transferred back into the bottle for storage.

#### Quantification of Nonmineralized Polyester- and Cellulose-Added ^13^C in Compost, ^13^C_nonmineralized_

For set 1, aliquots of 9 g freeze-dried compost per sample were milled for 2 min (IKA A11 basic). The compost aliquots from incubations with ^13^C-labeled PBAT and PBSeT were additionally homogenized by adding chloroform, followed by 30 min sonication and subsequent chloroform removal by evaporation—a treatment that dissolved any residual polyester particles and ensured uniform distribution of the dissolved polyester within the aliquot.^31^ These aliquots were then each milled a second time for 60 s (Retsch MM400; 60 Hz). Duplicate ∼4 mg subaliquots per ∼9 g aliquot were weighed into tin capsules for EA-IRMS analysis.

For ^13^C-labeled PBAT, this homogenization procedure was insufficient to achieve reproducible results (based on first analyses, data not shown). Therefore, for ^13^C-labeled PBAT samples, the remaining compost material in each incubation bottle was homogenized by adding chloroform directly into the bottle, followed by sonication for 30 min with periodic swirling. Following removal of chloroform by evaporation, the dry homogenized sample was stirred by spatula and a ∼9 g compost aliquot was taken directly from the bottle by spatula. The aliquot was milled for 2 min, and duplicate ∼4 mg subaliquots were weighed into tin capsules for EA-IRMS analysis. Data obtained using this adjusted homogenization procedure for ^13^C-labeled PBAT are presented herein.

The sample aliquots in the tin capsules were analyzed using an elemental analyzer (FlashEA 1112, Thermo Fisher) connected through a continuous flow interface (Conflo IV, Thermo Fisher) to an isotope ratio mass spectrometer (Delta V Plus, Thermo Fisher) (EA-IRMS). The EA-IRMS was operated and calibrated as described in Section S6, Supporting Information. Calculations of nonmineralized substrate-derived ^13^C, ^13^C_nonmineralized_, in the compost were performed as previously described^14,31^ and are presented in Section S6, Supporting Information. In brief, we determined carbon contents and δ^13^C values of the analyzed samples using calibration curves based on organic compounds of known carbon contents and ^13^C-isotopic compositions, including ^13^C-enriched glucose standards. Sample δ^13^C values were then converted to atom % ^13^C and the amounts of nonmineralized ^13^C from ^13^C-labeled polyesters and cellulose were subsequently calculated.

#### Quantification of Residual Polyesters, ^(13)^C_residual polyester_

Prior to analyzing samples from the two sets of compost incubation experiments, we developed and validated the method to solvent extract and quantify residual polyester from compost, based on previously validated protocols for soils^34,35^ (see Section S8, Supporting Information for details). The refined extraction and quantification protocols used for set 1 and set 2 samples are detailed in Section S9, Supporting Information.

In brief, set 1 subsamples (i.e., duplicate ∼9 g compost aliquots per incubation bottle, including aliquots from blank compost controls) were pre-extracted for 60 min with MeOH to remove most of the extractable COM (without extracting or chemically altering the polyesters; see Section S8, Supporting Information) before extraction of residual polyester with 9:1 (v/v) CHCl_3_:MeOH for 60 min (>20 extraction cycles; R306S heating apparatus with EZ30H extractor chamber, Behr LaborTechnik). The removal of co-extractable COM by pre-extraction was required because of the low concentrations of the ^13^C-labeled polyesters. Set 2 subsamples (i.e., single ∼15–20 g aliquots per incubation bottle, not including blank compost samples) were not pre-extracted with MeOH (due to their higher polyester concentrations) but instead directly extracted with 9:1 (v/v) CHCl_3_:MeOH mixture for 20 extraction cycles (UniversalExtractor E-800, Büchi, Switzerland). Set 1 samples containing ^13^C-cellulose were not extracted due to the lack of a suitable solvent. Solvent extracts of set 1 samples were reduced in volume to a few milliliters by evaporation on the heating apparatus of the Soxhlet unit, followed by evaporating residual solvent at room temperature (∼21 °C) in a fume hood overnight. The dried extracts were then reconstituted in CDCl_3_ with 1,4-dinitrobenzene as internal quantification standard. For set 2 extracts, extract aliquots of known volume were dried overnight. The dried extracts were subsequently reconstituted in deuterated chloroform (CDCl_3_) with 1,4-di­(trifluoromethyl)­benzene as internal standard. All samples were analyzed on a Bruker Avance III 400 MHz NMR spectrometer equipped with a 5 mm BBFO Z-Gradient probe. All details pertaining to the ^1^H NMR analyses are provided in Section S7, Supporting Information.

## Results and Discussion

### Polymer Mineralization Dynamics

Mineralization extents, ^(13)^C_mineralized_, and corresponding mineralization rates of ^13^C-labeled PBAT, PBSeT, and cellulose (set 1), and of nonlabeled PBAT, PBSeT, and PHBH (set 2) are shown in Figure 1 (extents) and Section S10, Supporting Information (rates). ^(13)^C_mineralized_ values are expressed as the amounts of formed ^13^CO_2_ or CO_2_ in percent of the initially added polyester and cellulose ^13^C or total C. This normalization allows comparing the mineralization dynamics across the two experimental sets despite their different initial substrate concentrations (i.e., ∼5 mg ^13^C-labeled polyester or ^13^C-labeled cellulose (g dry compost)^−1^ for set 1 vs ∼100 mg unlabeled polyester substrate for set 2).

**Figure 1:**
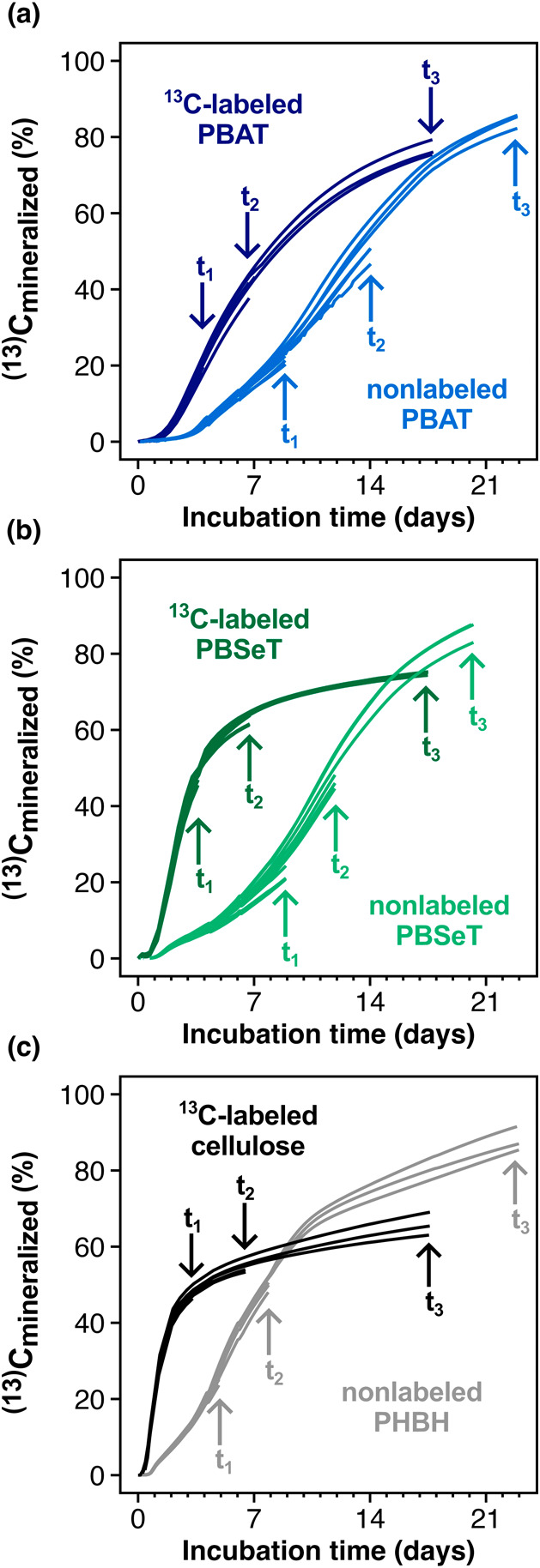
Cumulative mineralization extents of ^13^C-labeled substrates (set 1) and nonlabeled polyesters (set 2) in compost incubations. The tested polyesters were ^13^C-labeled and nonlabeled poly­(butylene adipate-*co*-terephthalate) (PBAT), ^13^C-labeled and nonlabeled poly­(butylene sebacate-*co*-terephthalate) (PBSeT), as well as the positive biodegradation controls ^13^C-labeled cellulose (set 1) and nonlabeled poly­(3-hydroxybutyrate-*co*-3-hydroxyhexanoate) (PHBH; set 2). The cumulative mineralization extents, ^(13)^C_mineralized_, of substrate carbon conversion into ^(13)^CO_2_ were obtained by integration of the respective mineralization rates and are expressed in percent of the mass of substrate-^(13)^C initially added to the incubations. Lines represent mineralization dynamics of individual incubation bottles and are plotted by linear interpolating between data points measured at a frequency of approximately 8 h. Vertical arrows indicate the times t_1_–t_3_ at which groups of triplicate incubations were terminated for determination of nonmineralized polymer-derived ^13^C (set 1) and residual polyester remaining in the compost (sets 1 and 2). For set 1, groups of triplicate incubations were terminated at incubation times t_1_ = 4 days, t_2_ = 7 days, and t_3_ = 18 days (panels a, b, and c). For the incubations with nonlabeled polyesters (set 2), three groups were terminated when mineralization extents reached approximately 25%, 50%, and 90%, which corresponded to t_1_ = 9 days, t_2_ = 14 days, and t_3_ = 23 days for PBAT (panel a), t_1_ = 9 days, t_2_ = 12 days, and t_3_ = 20 days for PBSeT (panel b), and t_1_ = 5 days, t_2_ = 8 days, and t_3_ = 23 days for PHBH (panel c).

Mineralization dynamics of replicate incubation bottles were in good agreement within both sets, demonstrating that the incubation setup coupled to the respirometric analysis of ^13^CO_2_ and ^12^CO_2_ (set 1) and of total CO_2_ (set 2) resulted in highly reproducible measurements.

#### ^13^C-Labeled PBAT, PBSeT, and Cellulose (Set 1)

The mineralization curves of the ^13^C-labeled polyesters and ^13^C-labeled cellulose displayed sigmoidal shapes with an initial lag phase (duration of 1–2 days), followed by a second phase of high mineralization rates and steep increases in ^13^C_mineralized_ (between approximately days 3 and 7 of incubation), and a third final phase in which mineralization rates slowed down and ^13^C_mineralized_ leveled off (Figure 1; Section S10, Supporting Information).

The initial lag phases likely reflect microbial adaptation to the added substrates.^36^ The adaptation may have included the time required for microbial degraders to colonize the substrate surfaces and/or to upregulate production and secretion of extracellular esterases and cellulases needed to cleave the substrate structures into oligomers and monomers small enough for subsequent microbial uptake and utilization. Such initial lag phases are commonly observed in polyester biodegradation experiments, including compost incubations.^11–13,37^ The second phase, characterized by near-linear increases in ^13^C_mineralized_, represents the most active phase of substrate turnover. The subsequent slowdown of mineralization rates in the third phase likely reflects depletion of polyesters and cellulose available for microbial utilization. Although only ∼60% of the ^13^C-labeled PBAT and ^13^C-labeled PBSeT (and ∼50% of the ^13^C-cellulose) had mineralized to ^13^CO_2_ at the onset of the third phase, compost solvent extraction confirmed low residual polyester amounts at this point and thus that the slowdown in mineralization was caused by substrate depletion (see detailed discussion below). The ^13^CO_2_ formed in the third phase thus likely had significant contributions from the turnover of substrate-derived ^13^C that had been incorporated into microbial biomass in the second phase of incubation.

When comparing mineralization rates across substrates, ^13^C-labeled PBSeT mineralized faster up to the end of the second phase, and the ^13^C_mineralized_ curves showed a sharper transition into the third phase as compared to those of ^13^C-labeled PBAT (Figure 1a,b). A similar trend was also observed for ^13^C-cellulose (Figure 1c). These observations suggest that mineralization rates of ^13^C-PBSeT and ^13^C-cellulose were much higher than mineralization rates of substrate-^13^C incorporated into microbial biomass. We note that ^13^C-labeled PBAT and ^13^C-labeled PBSeT were both labeled in B, ruling out that the position of the label caused differences in ^13^CO_2_ formation dynamics.

Interestingly, the final mineralization extents at 18 days of incubation were slightly higher for ^13^C-labeled PBAT than for ^13^C-labeled PBSeT despite the latter showing higher mineralization rates in the second phase. This finding may reflect continuous mineralization of residual ^13^C-labeled PBAT in both the second and third phases, whereas ^13^CO_2_ formation in the third phase of PBSeT incubations was dominated by mineralization of biomass-incorporated ^13^C. In all cases, mineralization was still ongoing at the final sampling time point t_3_, implying that higher mineralization extents would have been achieved if incubations had been continued.

#### Nonlabeled PBAT, PBSeT, and PHBH (Set 2)

Similar to the ^13^C-labeled substrates, mineralization curves of the nonlabeled polyesters show characteristic sigmoidal shapes: an initial lag-phase of a few days characterized by slow mineralization, a subsequent second phase with steep increases in C_mineralized_, and a final third phase in which mineralization slowed down and C_mineralized_ leveled off (Figure 1). The transition into the third phase was sharp and most distinct for PHBH (at around 9–10 days of incubation) and more gradual for PBSeT and PBAT. The three distinct phases are also apparent from the corresponding measured mineralization rate data (Section S10, Supporting Information).

When comparing the nonlabeled polyesters, mineralization rates during the first two phases were highest for PHBH, consistent with it serving as a positive biodegradation control, followed by PBSeT and finally PBAT. Yet, after 3 weeks of incubation, C_mineralized_ values of the three polyesters were comparable at 80% to 90%. At the final sampling time point t_3_, all mineralization curves still exhibited positive slopes (Figure 1), indicating continued mineralization of polyester-derived carbon, either from residual polyester and/or from turnover of microbial biomass formed from polyester C. Consequently, as for set 1, a higher final C_mineralized_ would have been obtained if the incubations had been extended.

#### Comparison of Mineralization Dynamics between Experimental Sets 1 and 2

Differences in the mineralization dynamics of PBAT and PBSeT between the two experiments require careful interpretation given that two different compost batches were used. Yet, both composts were obtained from the same vendor, had similar physicochemical properties (Section S3, Supporting Information), and displayed highly similar background mineralization (Section S11, Supporting Information), suggesting comparable microbial activities. Notably, the nonlabeled PBAT and PBSeT exhibited substantially longer initial lag phases (Figure 1) and lower relative mineralization rates during the second phase (Section S10, Supporting Information) as compared to their ^13^C-labeled counterparts. It is unlikely that these differences originated from differences in polyester monomeric composition given the similar T contents of the nonlabeled and ^13^C-labeled polyesters (see Materials and Methods). It is, however, possible that part of the differences in mineralization dynamics reflected the MW distributions of the polyesters (Section S2, Supporting Information): the nonlabeled polyesters contained a larger fraction of higher MW chains which potentially exhibited slower mineralization (Figure S2, Supporting Information). Yet, there was substantial overlap in the MW distributions between labeled and nonlabeled polyesters, suggesting that the different mineralization dynamics cannot be ascribed to the MW characteristics alone.

A plausible explanation for the slower mineralization rates of nonlabeled PBAT and PBSeT in set 2 as compared to set 1 is their higher initial concentrations in the compost. It is likely that the high initial polyester concentrations corresponded to an amount of polyester substrate that was in excess to the extracellular enzymatic and metabolic capacity of microorganisms in the compost, resulting in longer lag phases and slower mineralization rates. This interpretation suggests that mineralization rates determined in standardized compost biodegradability tests—commonly conducted at high initial polymer concentrations to generate sufficient polymer-derived CO_2_ to delineate it from high background mineralization of COM—may underestimate biodegradation rates compared to those observed at lower polyester concentrations more representative of most composting facilities.

Consistent with this assessment is the observation that differences in the mineralization dynamics of PBAT and PBSeT were less pronounced for the nonlabeled than for the ^13^C-labeled polyesters: high polyester concentrations in excess of the enzymatic and metabolic capacity of microbes may mask effects of polyester chemical composition on biodegradability. Studies are thus needed that systematically assess the effects of initial polyester concentration and polyester chemistry on absolute and relative biodegradation rates.

### Quantification and Characterization of the Nonmineralized Fraction

#### Quantification of Nonmineralized Polyester- and Cellulose-Added ^13^C, ^13^C_nonmineralized_ (= ^13^C_biomass_ + ^13^C_residual polyester_)

Figure 2a shows the amounts of nonmineralized polyester- and cellulose-added ^13^C that remained in the compost samples at their sampling times t_1_–t_3_, as determined by EA-IRMS. This analysis was limited to the ^13^C-labeled polyester and ^13^C-labeled cellulose samples (set 1) given that the ^13^C label is critical to distinguish added substrate-derived carbon from the carbon background in the compost in the EA-IRMS analysis. The ^13^C_nonmineralized_ may include both residual polyester- and cellulose-^13^C, ^13^C_residual polyester/cellulose_, as well as polyester- and cellulose-derived ^13^C that was incorporated into microbial biomass, ^13^C_biomass_.

**Figure 2:**
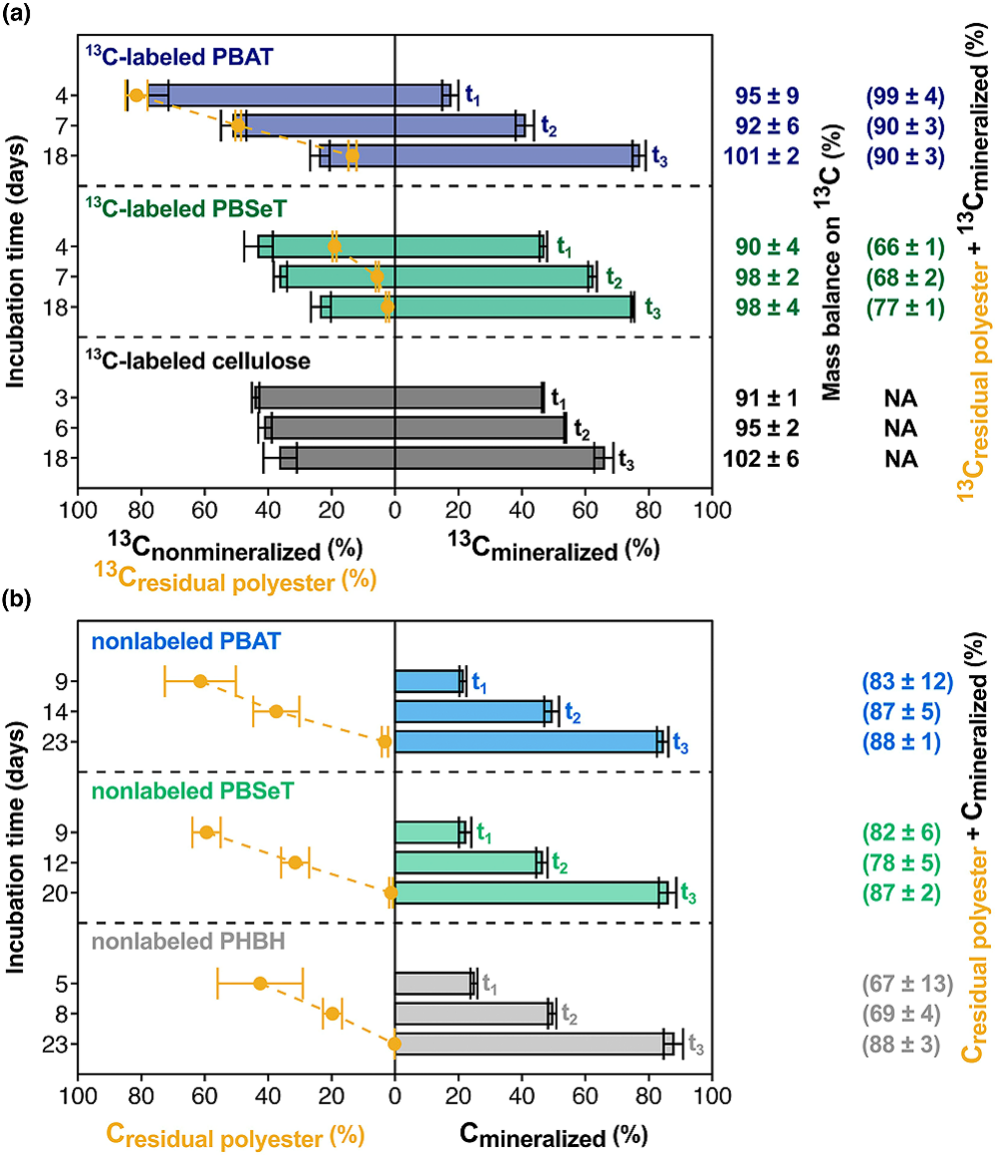
(a) Overview of the amounts of nonmineralized ^13^C (^13^C_nonmineralized_; bars to the left), residual polyester ^13^C (^13^C_residual polyester_; as orange circles), and mineralized ^13^C (^13^C_mineralized_; bars to the right with values replotted from Figure 1a,b) for set 1 incubations with ^13^C-labeled poly­(butylene adipate-*co*-terephthalate) (^13^C-labeled PBAT), ^13^C-labeled poly­(butylene sebacate-*co*-terephthalate) (^13^C-labeled PBSeT), and ^13^C-labeled cellulose. The amounts of residual cellulose in compost could not be determined due to the lack of a suitable extraction solvent. The three bars for each substrate correspond to the three sampling times t_1_, t_2_, and t_3_, with triplicate incubation bottles at each time. The mass balances on ^13^C over the course of the incubations are shown on the right and corresponds to the sum of ^13^C_nonmineralized_ and ^13^C_mineralized_ in percent of the total substrate-^13^C initially added to the compost. (b) Overview of the amounts of residual polyester C (C_residual polyester_; orange circles) and mineralized C (C_mineralized_; bars to the right with values replotted from Figure 1a,b) for set 2 incubations with nonlabeled PBAT, PBSeT, and poly­(3-hydroxybutyrate-*co*-3-hydroxyhexanoate) (PHBH). The three bars for each substrate correspond to the three sampling times t_1_, t_2_, and t_3_, with triplicate incubation bottles at each time. The sum of C_residual polyester_ and C_mineralized_ is shown on the right of both panels (a) and (b) in percent of the total substrate-C initially added to the compost. All values in panels (a) and (b) are averages of three triplicate incubation bottles per sampling time point t_1_, t_2_, and t_3_, and error bars represent the respective standard deviations.

As expected, ^13^C_nonmineralized_ in the compost decreased with increasing ^13^C_mineralized_ (Figure 2a). The ^13^C mass balance (i.e., sum of ^13^C_nonmineralized_ and ^13^C_mineralized_; the latter being replotted from data shown in Figure 1) for the two polyesters and cellulose ranged between 90 ± 4% and 102 ± 6% and, for most triplicate samples, did not differ from 100% within the errors of the analysis (numbers shown to the right of Figure 2a). Incubations with closed mass balances on added ^13^C imply that the ^13^C-labeling approach allowed for accurate tracking of polyester and cellulose ^13^C over the course of compost incubations, despite the comparatively low initial ^13^C-labeled polyester and cellulose concentrations and the high COM content and thus high background in EA-IRMS analysis. Slightly incomplete mass balances in some incubations likely reflected the difficulty of ensuring that the 4 mg subsamples analyzed by EA-IRMS were representative of the bulk compost sample. This finding underlines the importance of extensive homogenization of the compost prior to subsampling and analysis (see Materials and Methods).

#### Quantification of Residual Polyester, ^(13)^C_residual polyester_

The residual amounts of (^13^C-labeled) PBAT, (^13^C-labeled) PBSeT, and PHBH, ^(13)^C_residual polyester_, extracted from the samples of experimental sets 1 and 2 at sampling times t_1_ to t_3_ are shown in Figure 2 (orange symbols) and are expressed as extracted mass of polyester carbon in percent of the initially added polyester carbon mass. The standard deviations of C_residual polyester_ at the two intermediate time points were larger than those of the corresponding C_mineralized_ for nonlabeled PBAT (i.e., 11% vs 1% at t_1_ and 7% vs 2% at t_2_), PBSeT (5% vs 2% at t_1_ and 4% vs 2% at t_2_), and PHBH (13% vs 1% at t_1_ and 3% vs 1% at t_2_). These larger uncertainties likely reflect the general challenge of obtaining a representative compost subsample from the incubation bottles for C_residual polyester_ analysis due to the nonuniform distribution of residual polyester in the compost. Nonuniformity thus likely was not completely eliminated by the chosen compost homogenization treatment (see Materials and Methods).

Values of ^13^C_residual polyester_ for ^13^C-labeled PBAT and ^13^C-labeled PBSeT (set 1) decreased with increasing ^13^C_mineralized_ from t_1_ to t_3_ (Figure 2a). For ^13^C-labeled PBAT, the sum of ^13^C_residual polyester_ and ^13^C_mineralized_ amounted to 90% to 99% of the added PBAT-^13^C. Considering that mass balances on ^13^C were (almost) closed, we consider that the remaining 1–10% of the added PBAT-^13^C had been incorporated into microbial biomass (see details below). By comparison, for ^13^C-labeled PBSeT, the sum of ^13^C_residual polyester_^13^C_mineralized_ amounted to 66–77% of the added PBSeT-^13^C, implying that a larger fraction of added ^13^C was incorporated into microbial biomass (approximately 23–34%; see details below).

Similar to the residual ^13^C-labeled polyesters, C_residual polyester_ of nonlabeled PBAT, PBSeT, and PHBH (set 2) decreased with increasing C_mineralized_ from t_1_ to t_3_. The sum of C_residual polyester_ and the respective C_mineralized_ accounted for 78–88% of added PBAT and PBSeT carbon and for 67–88% of the added PHBH carbon. While mass balances could not be determined for set 2 incubations given that nonlabeled polyesters were used, we consider them closed (given that a similar experimental setup was used) and consider the ‘missing’ 12–22% of the added PBAT and PBSeT carbon and 12–33% of the PHBH carbon to have been incorporated into microbial biomass. Notably, the estimated C_biomass_ decreased with increasing incubation time, suggesting that microbial biomass built from polyester-derived carbon itself was remineralized to CO_2_ in later stages of the incubation, as discussed further below.

#### Characterization of Extracted Residual PBAT and PBSeT

In addition to quantifying residual PBAT and PBSeT extracted from the compost, the ^1^H NMR spectra allow to determine their monomeric compositions (see Section S7, Supporting Information). Figure 3 shows that the T contents of extracted residual ^13^C-labeled and nonlabeled PBAT and PBSeT increased with decreasing residual polyester amounts and thus with increasing extents of polyester biodegradation. This increase was most pronounced for ^13^C-labeled PBAT and ^13^C-labeled PBSeT (set 1), with T contents increasing from an initial 46 and 47 mol % (open circles) to approximately 66 ± 1% and 75 ± 6% after 18 days of incubation, respectively (Figure 3a). Increases in T content were less pronounced in incubations of nonlabeled PBAT and PBSeT (set 2) (Figure 3b).

**Figure 3:**
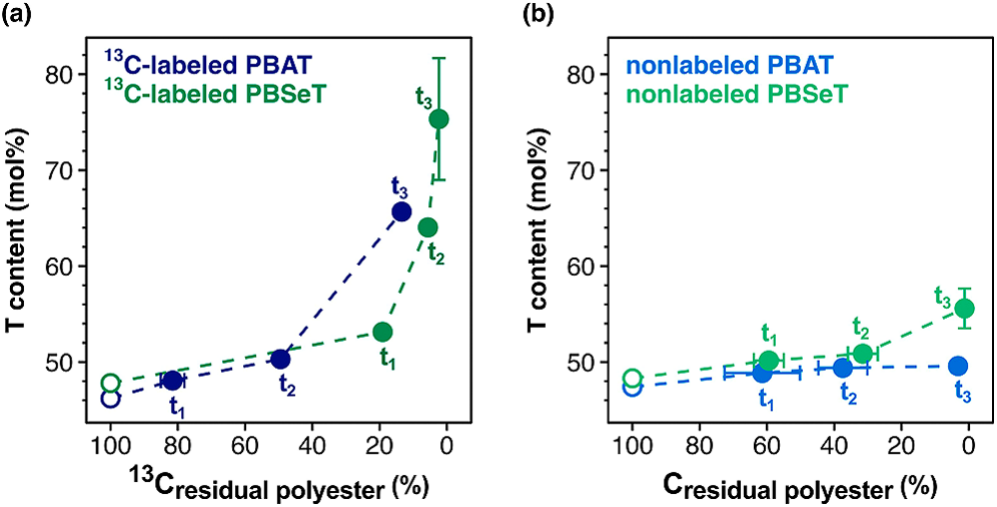
Terephthalate (T) contents in residual (a) ^13^C-labeled poly­(butylene adipate-*co*-terephthalate) (PBAT) and ^13^C-labeled poly­(butylene sebacate-*co*-terephthalate (PBSeT) (set 1) and (b) nonlabeled PBAT and PBSeT (set 2) extracted from compost samples removed from the incubators at three incubation times t_1_, t_2_, and t_3_. T contents are expressed in mol % of the total diacids in PBAT (i.e., T in mol % of the sum of T and adipate (A)) and PBSeT (i.e., T in mol % of the sum of T and sebacate (Se)). The T contents are plotted versus residual amounts of polyester ^(13)^C, ^(13)^C_residual polyester_, expressed in percent of the initially added polyester ^(13)^C. Data points and error bars represent means and standard deviations, respectively, of triplicate samples. The open symbols correspond to the initial T contents of the polyesters (i.e., at C_residual polyester_ = 100%).

The increases in T contents can be ascribed to crystalline domains in PBAT and PBSeT being enriched in T, while amorphous domains are enriched in A and Se. Preferential hydrolysis of esters in the amorphous over crystalline domains then results in increased T contents of residual PBAT and PBSeT, as previously reported in both enzymatic hydrolysis and soil biodegradation studies.^14,38–42^ The data suggest that increases in T contents are particularly pronounced when biodegradation rates are high (i.e., for ^13^C-labeled polyesters in set 1), possibly reflecting that the rates of enzymatic hydrolysis control overall biodegradation. Conversely, lower rates of biodegradation at higher initial polyester concentrations (i.e., here for nonlabeled polyesters in set 2) may have been rate-controlled by the adaptation and growth of microbial degraders. In any case, the increases in T contents of the residual polyesters are expected to slow enzymatic hydrolysis of the residual polyesters.^40^ The increased T contents in the residual may thus have contributed to the slowdown in polyester mineralization from the second to the third phase of the incubations, besides the slowdown in mineralization that resulted from depletion of residual polyester and the slow turnover of polyester-derived carbon incorporated into the microbial biomass.

### Incorporation of Polymer-^(13)^C into Microbial Biomass

We estimated the extents of biomass incorporation of polyester-^(13)^C, ^(13)^C_biomass_, in incubations of both experimental sets by subtracting the sum of ^(13)^C_mineralized_ and ^(13)^C_residual polyester_ from the initially added amounts of polyester ^(13)^C (Figure 4). ^(13)^C_biomass_ is expressed as a percent of the total added amounts of polyester-^(13)^C. We chose this approach as it applies to both experimental sets. For ^13^C-labeled polyesters in set 1, ^13^C_biomass_ was additionally estimated by subtracting ^13^C_residual polyester_ from ^13^C_nonmineralized_. The two approaches generally yielded comparable results for set 1 incubations (Section S12, Supporting Information). This approach to estimate C_biomass_ is considered robust as residual polyesters are quantitatively extracted and quantified (Section S8, Supporting Information). While extraction efficiencies may be lower for low-molecular-weight monomers and oligomers given their charge and polarity, we do not expect these to have been present in substantial amounts as they were shown to rapidly and extensively biodegrade even in less active soils.^31,43^

**Figure 4:**
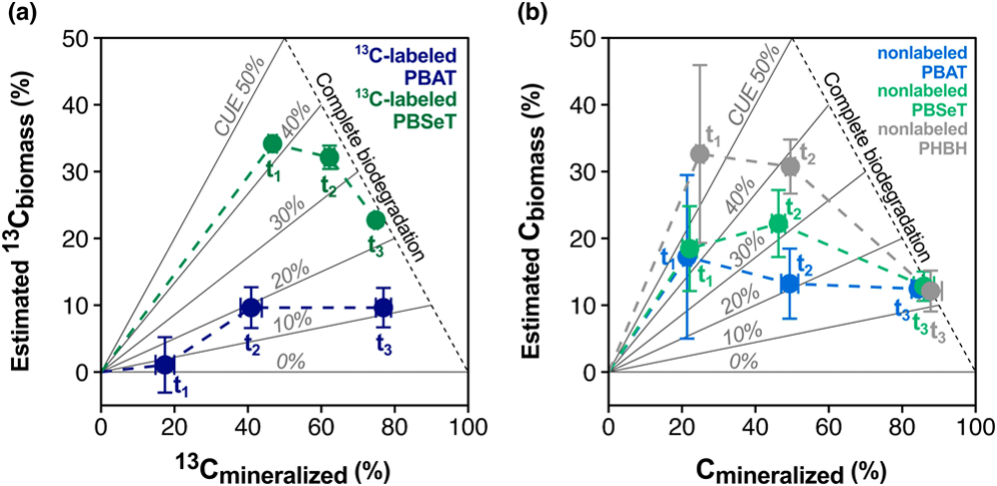
Estimates of amounts of polyester carbon incorporation into microbial biomass, C_biomass_, for (a) ^13^C-labeled poly­(butylene adipate-*co*-terephthalate) (PBAT) and ^13^C-labeled poly­(butylene sebacate-*co*-terephthalate) (PBSeT) (set 1) and (b) for nonlabeled PBAT, PBSeT, and poly­(3-hydroxybutyrate-*co*-3-hydroxyhexanoate) (PHBH) (set 2). The polyester carbon mineralization extents, C_mineralized_, on the *x*-axis are replotted from Figure 1 and C_biomass_, and the *y*-axis values were calculated as described in the main text. Both carbon pools are expressed in percent of the total amounts of polyester carbon initially added to the incubations. The series of gray lines display reference carbon use efficiencies (CUE), which correspond to the amounts of polyester carbon incorporated into microbial biomass in percent of the total amounts of microbially metabolized polyester carbon (i.e., the sum of biomass incorporated and mineralized carbon amounts). The dashed line corresponds to different incubation outcomes with complete polyester biodegradation (i.e., different possible combinations of C_biomass_ and C_mineralized_ that correspond to 100% of added polyester ^(13)^C). Incubations with nonlabeled polyesters (panel b) were conducted at approximately 20-fold higher initial polyester concentrations than in incubations with ^13^C-labeled polyesters (panel a).

Calculated ^(13)^C_biomass_ values are plotted against the corresponding ^(13)^C_mineralized_ values at sampling times t_1_ to t_3_ (Figure 4). Gray solid lines indicate reference carbon use efficiency (CUE) values (i.e., the amount of polyester-C incorporated into biomass divided by the sum of amounts of polyester-C incorporated into biomass and polyester-C mineralized; CUE = ^(13)^C_biomass_ (^(13)^C_biomass_ + ^(13)^C_mineralized_)^−1^) (Section S12, Supporting Information, for calculated CUE values). The dashed diagonal line represents complete polyester biodegradation (i.e., the sum of ^(13)^C_biomass_ and ^(13)^C_mineralized_ corresponds to 100% of the initially added polyester ^(13)^C).

Figure 4a shows that biodegradation of ^13^C-labeled PBAT resulted in ^13^C incorporation into biomass, but that the resulting ^13^C_biomass_ was comparatively small, corresponding to estimated CUEs of only 6% at t_1_ and 19 and 11% at t_2_ and t_3_, respectively. For ^13^C-labeled PBSeT, ^13^C_biomass_ values were much higher with estimated CUEs of ∼42% and 34% at t_1_ and t_2_, respectively. The lower CUE of ∼23% at t_3_ implies that part of the previously biomass-incorporated ^13^C was mineralized to ^13^CO_2_. In fact, at t_3_, biodegradation of PBSeT was almost complete, as indicated by the sum of ^13^C_biomass_ + ^13^C_mineralized_ being close to 100% (i.e., the dashed diagonal line). The higher CUEs of ^13^C-labeled PBSeT than ^13^C-labeled PBAT may reflect that the former biodegraded more rapidly and thus that the supply rate of polyester carbon to microorganisms was higher, resulting in a metabolic shift toward anabolism. For nonlabeled polyesters, which were incubated at approximately 20-fold higher concentrations than the ^13^C-labeled polyesters, estimated CUE values for all three polyesters were between 40 to 55% at t_1_, albeit with larger variations between replicates (Figure 4b). CUEs decreased with increasing mineralization extent to ∼12–13% at t_3_ when biodegradation was close to complete for all polyesters.

The estimated C_biomass_ and corresponding CUE values have several important implications for the assessment of polyester biodegradation. First, intermediate to high CUE values determined herein indicate that a substantial fraction of polyester carbon metabolized by microorganisms in compost can be incorporated into microbial biomass. The maximum CUE of ∼50–60% estimated herein (i.e., approximately half of the metabolized polyester carbon was used for microbial biomass formation) is in good agreement with the maximum CUE values that were reported to be theoretically and experimentally possible for microbial utilization of organic substrates.^44,45^ Second, considering only mineralization extents in the assessment of PBAT and PBSeT biodegradation would have substantially underestimated their actual biodegradation extents. While we would have underestimated biodegradation over the entire incubation period, the implications would have been most significant when incubations were terminated: while none of the incubations in sets 1 and 2 had reached complete mineralization (i.e., ^13^C_mineralized_ remained below 100%) at t_3_, residual polyester amounts (i.e., ^(13)^C_residual polyester_) were close to 0% for all incubations except for ^13^C-labeled PBAT (with ^13^C_residual polyester_ ≈ 10% at t_3_ when ^13^C_mineralized_ was ∼80%). The importance of accounting for C_biomass_ is exemplified by ^13^C-labeled PBSeT: after 4 days, total biodegradation of ^13^C-labeled PBSeT (i.e., ^13^C_biomass_ + ^13^C_mineralized_) reached ∼81% and thus was substantially higher than ^13^C_mineralized_ of 47 ± 1%. At the final sampling time point at 18 days, biodegradation of ^13^C-labeled PBSeT was almost complete: ^13^C_residual polyester_ amounted to only 2 ± 1% while ^13^C_biomass_ was 23 ± 1%. At the same time, ^13^C_mineralized_ at t_3_ was ‘only’ 75 ± 1% and thus substantially below 100%. Without the quantification of ^13^C_residual polyester_ and ^13^C_biomass_, the incomplete mineralization could have been misinterpreted as a large fraction of 25% of the initially added ^13^C-labeled PBSeT remaining in the compost. Third, in all incubations ^(13)^C_biomass_ and thus ‘apparent’ CUEs decreased from t_2_ to t_3_, implying that microbial biomass formed from polyester ^(13)^C eventually underwent mineralization to ^(13)^CO_2_. This supports that the transition from the second to the third phase in the mineralization curves (Figure 1) corresponded to a transition from ^(13)^CO_2_ being formed primarily from direct microbial respiration of polyester carbon to being formed through turnover of polyester-carbon containing biomass. The estimated CUE values thus need to be considered ‘apparent’ as ^(13)^C_biomass_ is itself a dynamic pool. Extending the analysis from only mineralization data to also quantifying residual polyester C and estimating polyester-derived biomass C provides a more holistic interpretation of polyester biodegradation dynamics.

## Implications

The use of ^13^C-labeled polyesters in compost incubations in combination with the analysis of residual polyesters allows for (i) quantitative tracking of polyester ^13^C into both mineralized and nonmineralized ^13^C pools, (ii) closing mass balances on added ^13^C during and at the end of the incubations, (iii) determining the relative contribution of residual polyester to the nonmineralized ^13^C pool, and finally, (iv) estimating the extent of polyester-^13^C incorporation into microbial biomass during the biodegradation process—all while using initial polyester concentrations much lower and closer to reality than those used in standard biodegradability tests for industrial compost, as also carried out herein with nonlabeled polyesters. As such, this novel approach overcomes major interpretational ambiguities of standard compost biodegradability tests. We expect that this approach will not only be used for detailed mechanistic studies on polymer biodegradation—as done herein—but also for regulatory aspects to provide weight-of-evidence for polymer biodegradability assessment.^46,47^

We anticipate that the herein validated solvent extraction and ^1^H NMR quantification method will be routinely used as it does not require ^13^C-labeling. The method can be used to quantify residual polyesters both in laboratory compost incubations as well as in compost from industrial composting facilities and could readily be implemented into existing standard tests for certification. As recently demonstrated for soil-biodegradable mulch films,^34^ the chemical selectivity of ^1^H NMR spectroscopy allows to separately follow biodegradation of individual polyester components in commercial compostable plastic blends (e.g., PBAT and polylactic acid). While the approach does not provide information on the particle size of residual polyester(s) (given that these are dissolved during the extraction), the solvent extraction approach allows for high sample throughput, provides chemical selectivity, and accurately quantifies all residual polyester—and thus is advantageous for quantification over microscopy-based (microplastic) particle analyses that detect particles above a certain micrometer size threshold.

This study provides evidence that relative polyester biodegradation rates decrease with increasing initial polyester concentrations. Slowed biodegradation at high concentrations likely reflects that the added polyester mass exceeds the metabolic capacity of the compost microbial community, implying that the time required to achieve complete biodegradation in composting facilities as well as in standard biodegradation tests may increase with higher initial concentrations of compostable plastics at the start of the composting process. The use of ^13^C-labeled polyesters allows for running incubations at very low initial concentrations, eliminating this potential artifact.

This work demonstrates that a substantial carbon mass fraction of the tested polyesters—irrespective of the initial polyester concentration—is incorporated into microbial biomass and that the biomass itself is a dynamic carbon pool undergoing mineralization. The finding of high initial carbon use efficiencies around 50% implies that mineralization measurements can underestimate the extent of biodegradation by up to a factor of two. Significant parts of the nonmineralized polyester-added carbon in later stages of the incubation (when mineralization rates slow down and extents plateau) may thus no longer be present as residual polyester but instead as microbial biomass. Incomplete mineralization thus does not necessarily signify the formation of persistent polyester residues. Instead, a fraction of the nonmineralized polyester carbon may be present in the form of microbial biomass, which is an accepted biodegradation outcome. Using the approach presented herein, future studies can systematically assess how CUEs depend on polyester chemistry and polyester carbon supply rates to microorganisms during biodegradation.

## Supplementary Material



## Notes

S1—Chemicals; S2—Polyester synthesis and characterization; S3—Compost properties and preparation; S4—Overview of experimental setup and analytical workflow; S5—Quantification of mineralization rates and extents by ECHO-CRDS; S6—Quantification of nonmineralized polymer-derived ^13^C by EA-IRMS; S7—Polyester quantification by ^1^H NMR; S8—Quantification of residual polyester in compost by Soxhlet extraction coupled to quantitative ^1^H NMR: method development; S9—Quantification of residual polyester in compost by Soxhlet extraction coupled to quantitative ^1^H NMR: method application to incubation experiments; S10—Mineralization rates; S11—Blank compost CO_2_ formation; S12—Overview of biodegradation data (PDF)
