# Supplementary material for: Biodegradation of 13C‑Labeled and Nonlabeled Aliphatic–Aromatic Polyesters in Compost: Advancing Assessments through Combined Respirometry and Residual Polyester Analysis
Source: Environ Sci Technol. 2026 Jul 23;60(30):21190–200. doi: 10.1021/acs.est.6c00032 (PMC13449877; doi:10.1021/acs.est.6c00032)
Supplement: Supplementary file 1 [file es6c00032_si_001.pdf]

SUPPORTING INFORMATION

Biodegradation of ^{13}C- and Nonlabeled Aliphatic-Aromatic Polyesters in Compost: Advancing Assessments through combined Respirometry and Residual Polyester Analysis

Flora Wille[1†], Kevin Kleemann[1†], Yao Zhou[1], Madalina Jaggi[2], Stefano M. Bernasconi[2], Hannah Kleyer[3], Davide Ciccarese[3], Johannes Zimmer[3], Kai O. Siegenthaler[4], Martin Worch[4], Glauco Battagliarin[4], Andreas Künkel[4], Michael Sander[1,5*]

[1]Institute of Biogeochemistry and Pollutant Dynamics, Department of Environmental Systems Sciences, ETH Zürich, 8092 Zürich, Switzerland

[2]Geological Institute, Department of Earth and Planetary Sciences, ETH Zürich, 8092 Zürich, Switzerland

[3]Société des Produits Nestlé S.A., 1000 Lausanne, Switzerland

[4]Biodegradation and Microplastics Research, BASF SE, 67056 Ludwigshafen am Rhein, Germany

[5]Eawag, Swiss Federal Institute of Aquatic Science and Technology, Überlandstrasse 133, CH-8600 Dübendorf, Switzerland

[†] Authors contributed equally to the work

[*] Corresponding author:

Prof. Dr. Michael Sander; email: michael.sander@env.ethz.ch

Number of pages: 42

Number of figures: 16

Number of tables: 4

TABLE OF CONTENTS

SECTION S1 – Chemicals 3

SECTION S2 – Polyester synthesis and characterization 3

SECTION S3 – Compost properties and preparation 7

SECTION S4 – Overview of experimental setup and analytical workflow 8

SECTION S5 – Mineralization rate and extent quantification by ECHO-CRDS 9

Section S5.1 – Compost incubation system and CO_2 analysis 9
Section S5.2 – Mineralization calculations set 1 (^{13}C-labeled polyesters and cellulose) 10
Section S5.3 – Mineralization calculations set 2 (nonlabeled polyesters) 11

SECTION S6 – Quantification of nonmineralized substrate-derived ^{13}C by EA-IRMS 12

Section S6.1 – EA-IRMS calibration 12
Section S6.2 – EA-IRMS operation 12
Section S6.3 – Calculation of nonmineralized polymer-derived ^{13}C in compost 13

SECTION S7 – Polyester quantification by ^{1}H NMR 14

Section S7.1 – ^{1}H NMR sample preparation 14
Section S7.2 – NMR operation 15
Section S7.3 – Spectral processing 15
Section S7.4 – Chemical structures and ^{1}H NMR spectra of internal standards 15
Section S7.5 – Chemical structures and ^{1}H NMR spectra of pristine polyesters 16
Section S7.6 – ^{1}H NMR integrals for repeat unit quantification 18
Section S7.7 – Calculation of residual polyester mass and molar fractions 20

SECTION S8 – Quantification of residual polyester in compost by Soxhlet extraction coupled to quantitative ^{1}H NMR: method development 24

Section S8.1 – Soxhlet extraction procedure 24
Section S8.2 – Evaluation of background compost organic matter extracts and polyester concentrations 25
Section S8.3 – Effect of the number of soxhlet extraction cycles on polyester recoveries 28
Section S8.4 – Spike-recovery experiments 30
Section S8.5 – Note on limits of detection and quantification 32

SECTION S9 – Quantification of residual polyester in compost by soxhlet extraction coupled to quantitative ^{1}H NMR: incubation experiments 33

Section S9.1 – Set 1 (^{13}C-labeled polyesters) 33
Section S9.2 – Set 2 (nonlabeled polyesters) 35

SECTION S10 – Mineralization rates 36

SECTION S11 – Blank compost CO_2 formation 37

SECTION S12 – Overview of biodegradation data 38

REFERENCES 42

SECTION S1 – Chemicals

The purities and suppliers of chemicals used in this study are listed in **Table S1**.

Table S1. Overview of chemicals used, their purities, and suppliers.

Chemical	Purity (%)	Supplier
1,4-bis(trifluoromethyl)benzene	> 99.0%	TCI
1,4-dimethoxybenzene	> 99.0%	Acros Organics Chemicals
1,4-dinitrobenzene	98%	Sigma Aldrich
Chloroform (HPLC grade)	≥ 99.8	Sigma Aldrich
Chloroform-d (NMR grade)	99.8	Thermo Scientific
Methanol (HPLC grade)	≥ 99.9	Sigma Aldrich

SECTION S2 – Polyester synthesis and characterization

Polyester synthesis

The ^{13}C-labeled PBAT and ^{13}C-labeled PBSeT were synthesized from nonlabeled butanediol (B) and fully $^{13}C_4$-labeled B (CAS 286012-95-1, Sigma Aldrich, lot MBBB9482V), terephthalic acid (T), and adipic acid (A) or sebacic acid (Se), respectively, using established polycondensation protocols and a standard polymer synthesis apparatus. The synthesis reactions were carried out in a 250 mL stirred reactor equipped with a magnetic stirrer coupling. A representative image of the setup is provided in **Figure S1**.

Following the reaction, the polyester remaining in the flask was dissolved in dichloromethane and cast in its dissolved state onto a PTFE foil. After casting, the dichloromethane was evaporated under ambient conditions. Once the solvent was removed and the polyester had

solidified, the polyester was placed in a vacuum drying oven at 50 °C overnight to ensure complete removal of residual solvent.

Nonlabeled polyesters were received from BASF SE and stemmed from industrial scale production sites.

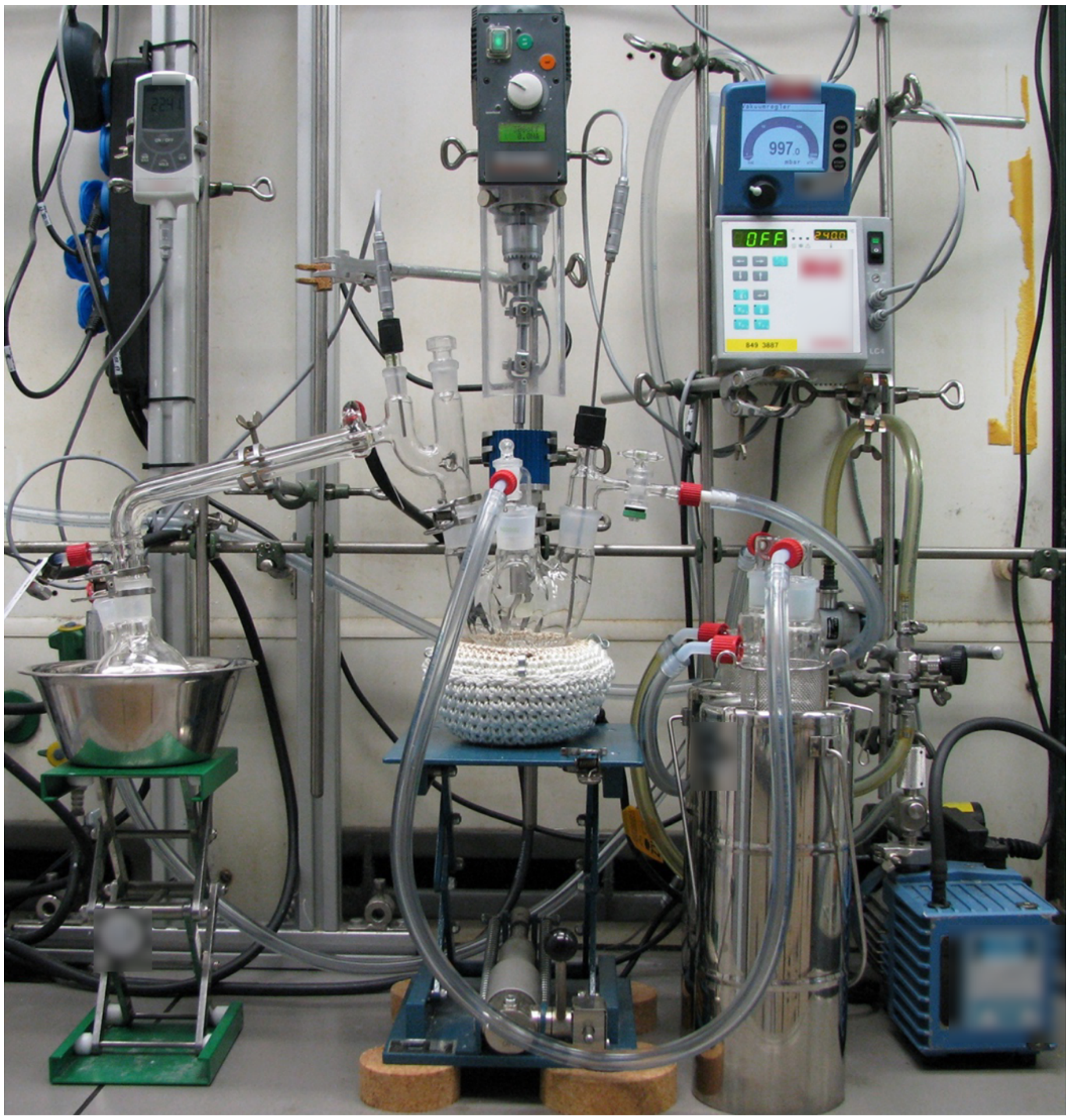

Figure S1. Laboratory setup for synthesis of ^{13}C-labeled polyesters.

Polymer characterization

The ^{13}C-labeled and nonlabeled PBAT and PBSeT variants were analyzed by size exclusion chromatography (SEC) using an Agilent 1100 Series instrument equipped with both a differential refractive index (dRI) detector and a UV detector set to a wavelength of 280 nm. The mobile phase was hexafluoroisopropanol (HFIP) containing 0.1 wt% potassium trifluoroacetate. Measurements were carried out at 35 °C with a volumetric flow rate of 1.0 mL min^{-1}. Sample solutions (1.5 mg mL^{-1}) were filtered through 0.2 µm Millex FG syringe filters before injection (50 µL).

The separation was performed on a column set consisting of a PL HFIPgel guard column (Agilent Technologies, 7.5 mm × 5 cm, Styrene-Divinylbenzene (SDV)) and two analytical PL HFIPgel columns (Agilent Technologies, 7.5 mm × 30 cm each). The first analytical column was packed with styrene-divinylbenzene (SDVB) gel with unknown exclusion limit, and the second with SDV gel with an exclusion limit of 10^2–10^5 g mol^{-1}.

Calibration was conducted using narrowly distributed poly(methyl methacrylate) (PMMA) standards (Polymer Standards Service) ranging from number average molecular weights Mn of 596 to 2’050’000 g mol^{-1}. Molar masses outside this range were extrapolated.

SEC results for commercial PBAT and PBSeT, as well as for ^{13}C-labeled PBAT and ^{13}C-labeled PBSeT, are shown in **Figure S2**. The y-axis represents the differential weight fraction per logarithmic molecular weight interval (dW $dlogM^{-1}$) for the analyzed sample. The entire curve thus corresponds to a full area normalized molecular weight distribution, where the area under the curve corresponds to the total polyester mass analyzed. The curves enable direct comparisons of the polyester mass distributions over the molecular weight ranges, independent of the exact mass of polyester analyzed.

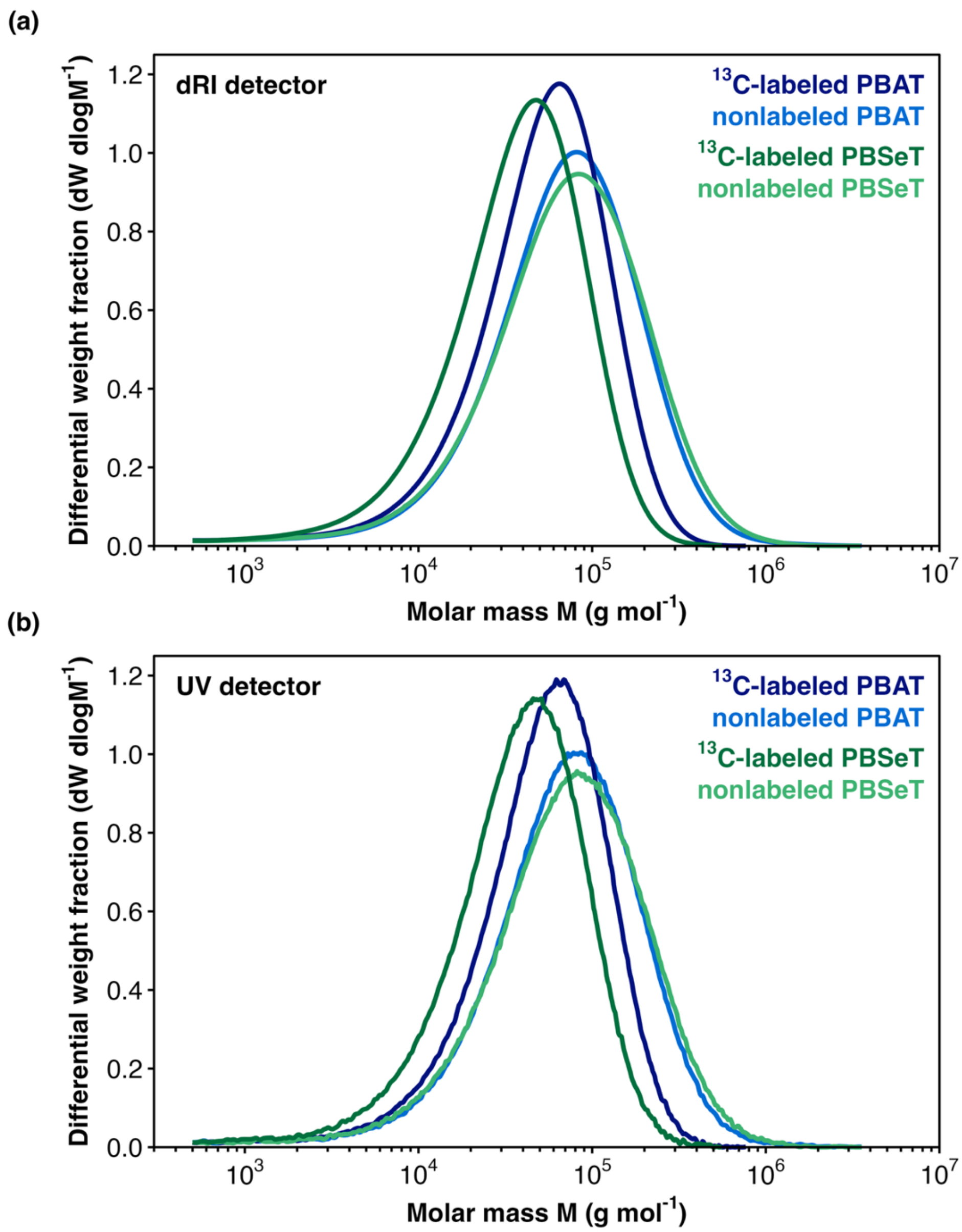

Figure S2. Molar mass distributions of ^{13}C-labeled and nonlabeled polyesters determined by size exclusion chromatography (SEC) with **(a)** a differential refractive index (dRI) detector and **(b)** a ultraviolet (UV) detector (set to 280 nm). The samples were ^{13}C-labeled poly(butylene adipate-co-terephthalate) (^{13}C-labeled PBAT, dark blue), ^{13}C-labeled poly(butylene succinate-co-sebacate-co-terephthalate) (^{13}C-labeled PBSeT, dark green), as well as commercial, nonlabeled PBAT (light blue), and commercial, nonlabeled PBSeT (light green).

SECTION S3 – Compost preparation and properties

For this study, a total of three batches of industrial compost were purchased from Normec OWS (Ghent, Belgium). Upon delivery, each compost batch was homogenized by dividing it into several portions, thoroughly mixing each portion in separate containers, and then recombining the portions. The received composts had water contents of ~48 wt%, as determined from the gravimetric mass loss of compost aliquots resulting from oven drying at 105 °C for 24 hours. Compost for extraction method development was stored at -20°C until being used.

The properties of each batch and their allocation to the corresponding experiments are summarized in **Table S2**.

Table S2. Physiochemical properties of the compost material from the three batches used in this study. All values are presented as reported by the supplier, Normec OWS.

Parameter	Batch 1*	Batch 2**	Batch 3***	Unit
Total solids (TS)	58.5	54.8	54.8	%
Volatile solids (VS)	41.7	39.5	39.5	% of TS
Ash content	58.3	60.5	60.5	% of TS
pH	8.0	8.1	8.1	-
Total N	29.1	19.5	19.5	g/kg TS
NH_4^+-N	<20.0	<20.0	<20.0	mg/L
NOx-N	368	500	500	mg/L
Salt content	3007	2888	2890	E.C., µS/cm
C/N	-	10	10	-

* used for method development (**Section S8**), received November 2023
** used for set 2 with nonlabeled polymers (**Section S9.2**), received April 2024
*** used for set 1 with ^{13}C-labeled polymers (**Section S9.1**), received May 2024

SECTION S4 – Overview of experimental setup and analytical workflow

A schematic overview of the experimental setup and the analytical workflow is provided in **Figure S3**.

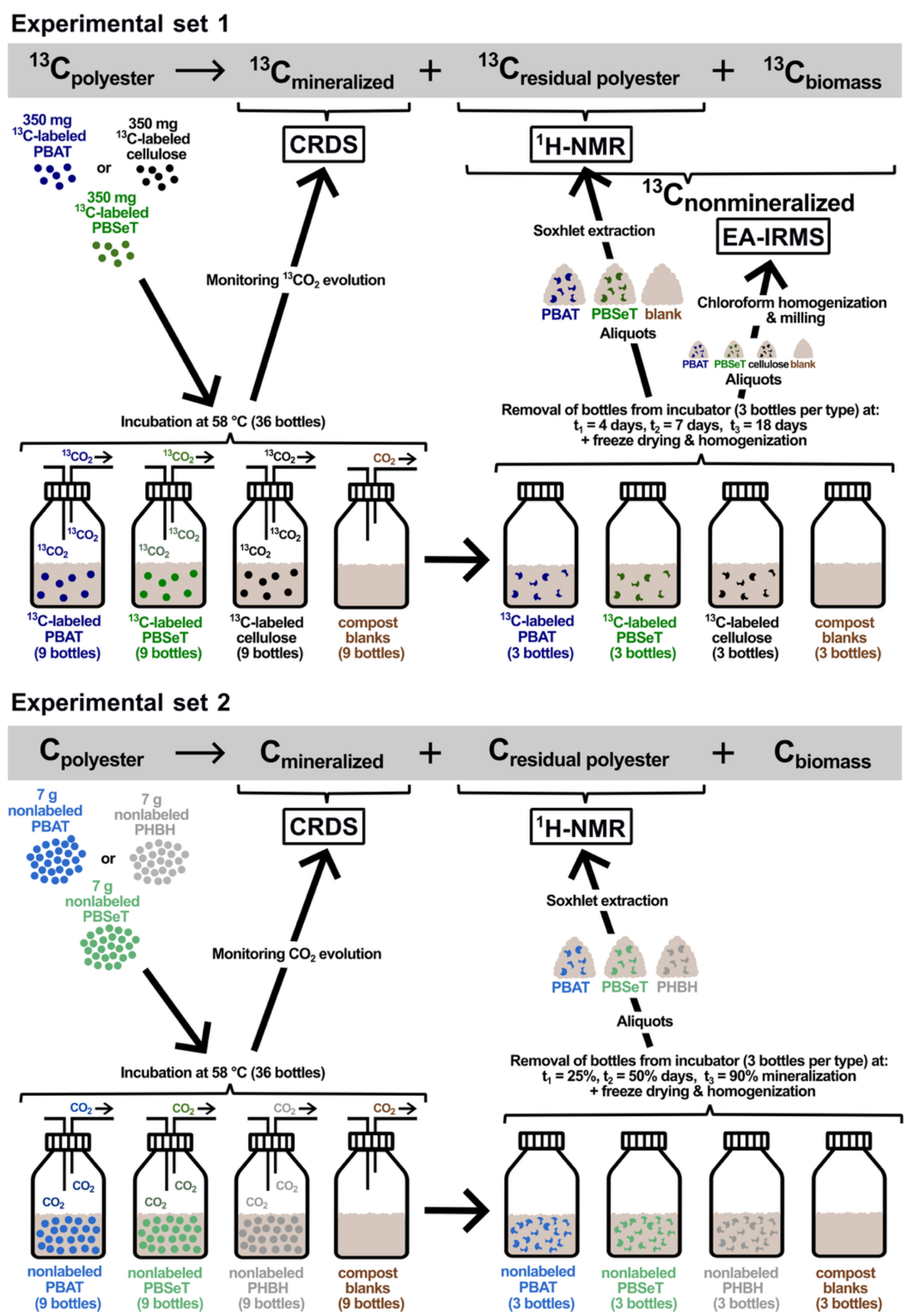

Figure S3. Schematic overview of the experimental setup and analytical workflow of experimental set 1 (^{13}C-labeled polyesters and celluose) and set 2 (nonlabeled polyesters). A detailed description is provided in the Materials and Methods section of the main text.

SECTION S5 – Mineralization rate and extent quantification by ECHO-CRDS

Section S5.1 – Compost incubation system and CO_2 analysis

A schematic representation of the respirometric compost incubation system employed in this study is presented in **Figure S4**. The setup included an incubator (ECHO; Respirometer ER Series, ECHO Instruments, Slovenia) connected to a cavity ring-down spectrometer (CRDS; G2201-i Isotopic Analyzer, Picarro, USA).

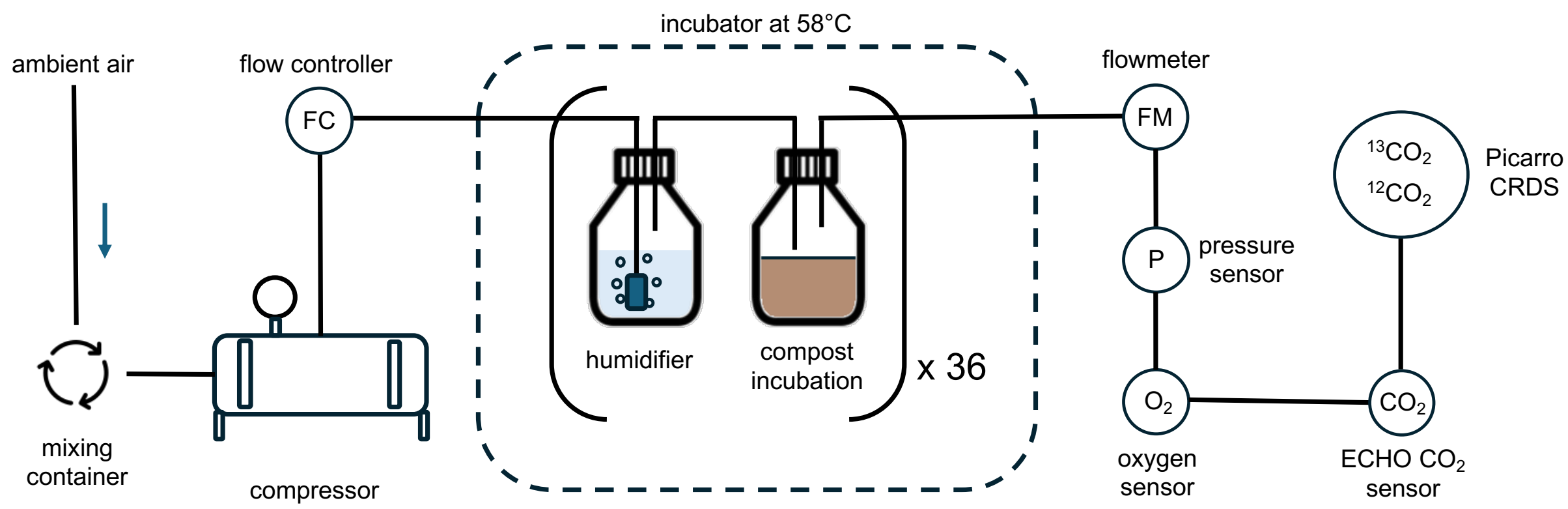

Figure S4. Schematic of the automated compost incubations system used for quantifying substrate mineralization to $^{(13)}CO_2$.

Ambient air was drawn from a mixed 1000 L intermediate bulk container —to ensure stable background CO_2 levels in the used air— into a compressor, which delivered the air to each incubation line. Flow rates were regulated by a mass flow controller (between 100-200 mL min^{-1}). The air in each line was first passed through a water-filled bottle with a submerged air dispenser to be humidified and thus minimize drying of the compost. The humidified air then was passed through the compost incubation bottle. The CO_2 concentration in the efflux gas of one compost incubation bottle at a time was determined by an IR-based CO_2 analyzer in the incubation units as well as by CRDS which was placed in series to the incubation-unit internal IR sensor. The CRDS quantified both $^{12}CO_2$ and $^{13}CO_2$ (and calculated the corresponding isotopic composition of CO_2

expressed in delta $(\delta)^{13}C$ values). The CRDS was calibrated periodically using two CO_2-in-air standards (i.e., 1000 ppm CO_2 at $\delta^{13}C$ = -38 ‰; 4000 ppm CO_2 at $\delta^{13}C$ = -3 ‰).

Section S5.2 – Mineralization calculations set 1 (^{13}C-labeled polyesters and cellulose)

The same equations were used for incubations containing ^{13}C-labeled polyesters and ^{13}C-labeled cellulose, which -hereafter- we refer to as 'substrate'. The procedure described below has previously been described and used.[1,2]

To quantify mineralization extents of ^{13}C-labeled substrates over time, we first quantified the fraction of CO_2 originating from the added substrate within the total compost CO_2 efflux. This was done by using the ^{13}C atom percentages of efflux CO_2 from compost samples with added substrate ($\%^{13}C_{compost+substrate}$), blank compost samples without added substrate ($\%^{13}C_{compost,\ blank}$), and the ^{13}C atom percentages of the substrates ($\%^{13}C_{substrate}$) as follows:

$$f_{substrate} = \frac{\%^{13}C_{compost+substrate} - \%^{13}C_{compost,\ blank}}{\%^{13}C_{substrate} - \%^{13}C_{compost,\ blank}} \qquad \textbf{Eq. S1}$$

The concentration of substrate-derived $^{13}CO_2$, $[^{13}CO_2]_{substrate}$ (μmol $^{13}CO_2$ mol_{air}^{-1}), in the bottle effluent gas at measurement time t was quantified by:

$$[^{13}CO_2]_{substrate}(t) = f_{substrate} \cdot [CO_2]_{compost+substrate}(t) \cdot \%^{13}C_{substrate} \qquad \textbf{Eq. S2}$$

where $[CO_2]_{compost+substrate}$ is the CO_2 concentration (μmol CO_2 mol_{air}^{-1}) in the effluent gas of compost incubation bottles with added substrate at measurement time t. Substrate ^{13}C-mineralization rates, $r_{substrate\text{-}^{13}C}$ (μg ^{13}C h^{-1}), at time t were then calculated by:

$$r_{substrate\text{-}^{13}C}(t) = \left[{}^{13}CO_2\right]_{substrate}(t) \cdot \frac{\dot{V} \cdot M}{V_m} \cdot 60\ \frac{min}{h} \qquad \textbf{Eq. S3}$$

where $\dot{V}$ is the volumetric gas flow rate through the incubation bottles (= 100 ± 5 mL min^{-1}), M is the molar mass of ^{13}C-labeled carbon (= 13.003 g mol^{-1}), and V_m is the molar volume of air at 25°C and 1 atm (= 22 465 mL mol^{-1}).

Integration of $r_{substrate\text{-}^{13}C}$ over time, assuming linear changes in rates between adjacent measurement time points, yielded the cumulative amount of substrate-^{13}C mineralized. Normalizing this value by the total substrate-^{13}C added to each incubation sample yielded mineralization extents, $^{13}C_{mineralized}$ (%), at time t:

$$^{13}C_{mineralized}(t) = \frac{\int_{t_0}^{t} r_{substrate}(t)\ dt}{m_{substrate} \cdot \%^{13}C_{substrate}} \cdot 100 \qquad \textbf{Eq. S4}$$

where $m_{substrate}$ is the added substrate mass (= 351 ± 1 mg), $\%^{13}C_{substrate}$ is the ^{13}C-mass percentage of the used substrates (7.5 and 7.2 wt% for PBAT and PBSeT, respectively).

Section S5.3 – Mineralization calculations set 2 (nonlabeled polyesters)

To quantify mineralization extents of nonlabeled substrates over time, the concentration of substrate-derived CO_2 , $[CO_2]_{substrate}$ (µmol CO_2 mol_{air}^{-1}), in the bottle effluent gas measured at time t was first quantified according to:

$$[CO_2]_{substrate}(t) = [CO_2]_{compost+substrate}(t) - [CO_2]_{compost,\ blank}(t) \qquad \textbf{Eq. S5}$$

where $[CO_2]_{compost+substrate}$ and $[CO_2]_{compost,\ blank}$ are the CO_2 concentrations (µmol CO_2 mol_{air}^{-1}) in the effluent gas of incubation bottles containing compost with and without (=blank) substrates, respectively. Mineralization rates, $r_{substrate}$ (µg carbon h^{-1}), at time t were then calculated using:

$$r_{substrate}(t) = [CO_2]_{substrate}(t) \cdot \frac{\dot{V} \cdot M}{V_m} \cdot 60\ \frac{min}{h} \qquad \textbf{Eq. S6}$$

where $\dot{V}$ is the volumetric gas flow rate through the incubation bottles (= 125 ± 5 mL min^{-1} until day 4 and 200 ± 5 ml min^{-1} thereafter), M is the molar mass of carbon (= 12.011 g mol^{-1}), and V_m is the molar volume of air at 25°C and 1 atm (= 22 465 mL mol^{-1}). Integration of $r_{substrate}(t)$ over time, assuming linear changes in rates between adjacent measurement timepoints, yielded the cumulative amount of carbon mineralized. Normalizing this value by the total substrate carbon added yielded mineralization extents, $C_{mineralized}$ (%), at time t:

$$C_{mineralized}(t) = \frac{\int_{t_0}^{t} r_{substrate}(t)\ dt}{m_{substrate} \cdot \%C_{substrate}} \cdot 100 \qquad \textbf{Eq. S7}$$

where $m_{substrate}$ is the added substrate mass (= 8.0 ± 0.1 g), $\%C_{substrate}$ is the carbon mass percentage of the used substrates (63, 66, and 57 wt% for PBAT, PBSeT, and PHBH, respectively).

SECTION S6 – Quantification of nonmineralized substrate-derived ^{13}C by EA-IRMS

Section S6.1 – EA-IRMS calibration

Linearity in the EA-IRMS response was established by analysis of standard compounds with known isotopic composition (i.e., nicotinamide with $\delta^{13}C$ = ca. -42 ‰ (Thermo), atropine with $\delta^{13}C$ = ca. -21 ‰ (Sigma), and glucose ($\delta^{13}C$ = ca. 103 ‰, prepared by mixing nonlabeled and $^{13}C_6$-labeled glucose (both from Sigma). The δ notation expresses the isotopic composition of a compound as its deviation, in parts per thousand (‰), from the carbon isotope ratio of the international Vienna Pee Dee Belemnite (VPDB) standard (i.e., $\delta^{13}C(VPDB)$ = 0 ‰).

Section S6.2 – EA-IRMS operation

The carbon isotopic signatures $\delta^{13}C$ and carbon contents (%C by mass) of the samples were determined using an elemental analyzer (FlashEA 1112, Thermo Fisher) coupled to a continuous flow interface (ConFlo IV) and an isotope-ratio mass spectrometer (Delta V Plus, Thermo Fisher) (EA-IRMS). Data were collected using Isodat (version 3.0), with helium as the carrier gas at a

flow rate of 80 mL min^{-1}. The tin capsules holding the compost samples were placed on a MAS 200 autosampler and sequentially introduced into the oxidative column of the EA, packed with chromium oxide and silvered cobaltous oxide and operating at 1020°C. Oxygen gas (O_2) was injected into the oxidative column for 3 seconds at a flow rate of 175 mL min^{-1} to facilitate combustion. The resulting gas was passed through a reductive column (containing elemental copper, operating at 650°C) and then through a drying column filled with magnesium perchlorate. A GC column (2 m, packed with Porapak QS 50/80 mesh, operating at 45°C) separated CO_2 from interfering gases before its introduction to the IRMS for isotopic composition analysis of the formed CO_2. The IRMS response was repeatedly calibrated using isotopic standards, as detailed above in **Section S6.1**.

Section S6.3 – Calculation of nonmineralized polymer-derived ^{13}C in compost

Calculations were conducted as previously published[1,2] and were performed identically to samples containing either polyester or cellulose. For simplicity, both are hereafter referred to as 'substrate'.

To quantify nonmineralized polymer-derived ^{13}C, we first calculated the fraction of carbon originating from the added substrate to the total measured compost carbon ($f_{substrate}$) by using **Eq. S1**, where $\%^{13}C_{compost+substrate}$, $\%^{13}C_{compost}$, and $\%^{13}C_{substrate}$ refer to the ^{13}C atom% of CO_2 from combustion of compost with added substrate, from compost without added substrate, and from only the substrate, respectively.

We subsequently calculated the mass of nonmineralized polymer-derived ^{13}C, $m(^{13}C_{nonmineralized})$ (µg ^{13}C), by multiplying the total carbon of the analyzed aliquot, $m(C_{compost+substrate})$ (µg C), by $f_{substrate}$ and the ^{13}C atom% of the added substrate, $\%^{13}C_{substrate}$, as well

as the ratio of the total compost mass in the incubation, m(compost$_{incubation}$) (g dry compost) to the mass of the compost aliquot analyzed using EA-IRMS, m(compost$_{EA\text{-}IRMS}$) (g dry compost):

$$m(^{13}C_{nonmineralized}) = m(C_{compost+substrate}) \cdot f_{substrate} \cdot \%^{13}C_{substrate} \cdot \frac{m(compost_{incubation})}{m(compost_{EA\text{-}IRMS})} \qquad \textbf{Eq. S8}$$

We then normalized the mass of nonmineralized substrate-derived ^{13}C to the mass of substrate-^{13}C added to each incubation bottle, m($^{13}C_{added}$) (µg ^{13}C), to obtain the percent of the added substrate-^{13}C that was nonmineralized in the compost at the end of the incubations, $^{13}C_{nonmineralized}$ (%):

$$^{13}C_{nonmineralized} = 100 \cdot \frac{m(^{13}C_{nonmineralized})}{m(^{13}C_{added})} \qquad \textbf{Eq. S9}$$

SECTION S7 – Polyester quantification by ^{1}H NMR

Section S7.1 – ^{1}H NMR sample preparation

Pure polyesters and polyester extracts obtained by Soxhlet extraction of compost samples were dissolved or reconstituted, respectively, in 100 mL round-bottom flasks (Lenz, DURAN®, NS 29/32) with 3 mL deuterated chloroform ($CDCl_3$) containing an internal standard at a concentration of approx. 2 mg mL^{-1}. As internal standards, either 1,4-bis(trifluoromethyl)benzene, 1,4-dimethoxybenzene, or 1,4-dinitrobenzene were used. Internal standards were selected based on their availability in our laboratory at time of reconstitution. For complete polyester dissolution or reconstitution, round-bottom flasks were sonicated in an ultrasonic bath for ~20 sec (VWR ultrasonic cleaner, model USC600D) while gently swirling. Aliquots of 1 mL were subsequently transferred to borosilicate NMR tubes (178 mm, Wilmad® Economy HTS), and the remaining ~2

mL were transferred to amber HPLC screw cap vials (BGB®, part no. 080401-W) and refrigerated for future analysis, if required.

Section S7.2 – NMR operation

Proton nuclear magnetic resonance (^{1}H NMR) spectra were recorded on a Bruker Avance III 400 MHz NMR instrument equipped with a Bruker 5 mm BBFO 400 MHz Z-gradient probe. The spectra were acquired in $CDCl_3$, with a pulse width of 4.62 μs, and a corresponding pulse power of 16.795 W. A total of 128 acquisition scans (NS) and 16 dummy scans (DS) were collected, with a relaxation delay (D1) of 15 s between scans. The acquisition time was 4.62 s per scan. Samples were continuously spun during acquisition.

Section S7.3 – Spectral processing

^{1}H NMR spectra were processed using Mnova version 15.0.1 (MestreLab) as follows: 1) chemical shifts (δ) were referenced to the ^{1}H signal of residual nondeuterated chloroform ($CHCl_3$) in $CDCl_3$ at $\delta = 7.26$ ppm, 2) the phase was manually adjusted, 3) the baseline was set to zero using a manual segmented linear correction, and 4) selected characteristic peaks used for polyester quantification, including peaks of the internal standards, were manually integrated. Annotated ^{1}H NMR spectra of the internal standards and polyesters used in this study are provided in **Sections S7.4** and **S7.5**, and the corresponding integral regions of the selected polyester peaks are detailed in **Section S7.6**.

Section S7.4 – Chemical structures and ^{1}H NMR spectra of internal standards

Figure S5 shows the chemical structures and annotated ^{1}H NMR spectra of the internal standards 1,4-bis(trifluoromethyl)benzene, 1,4-dimethoxybenzene, and 1,4-dinitrobenzene.

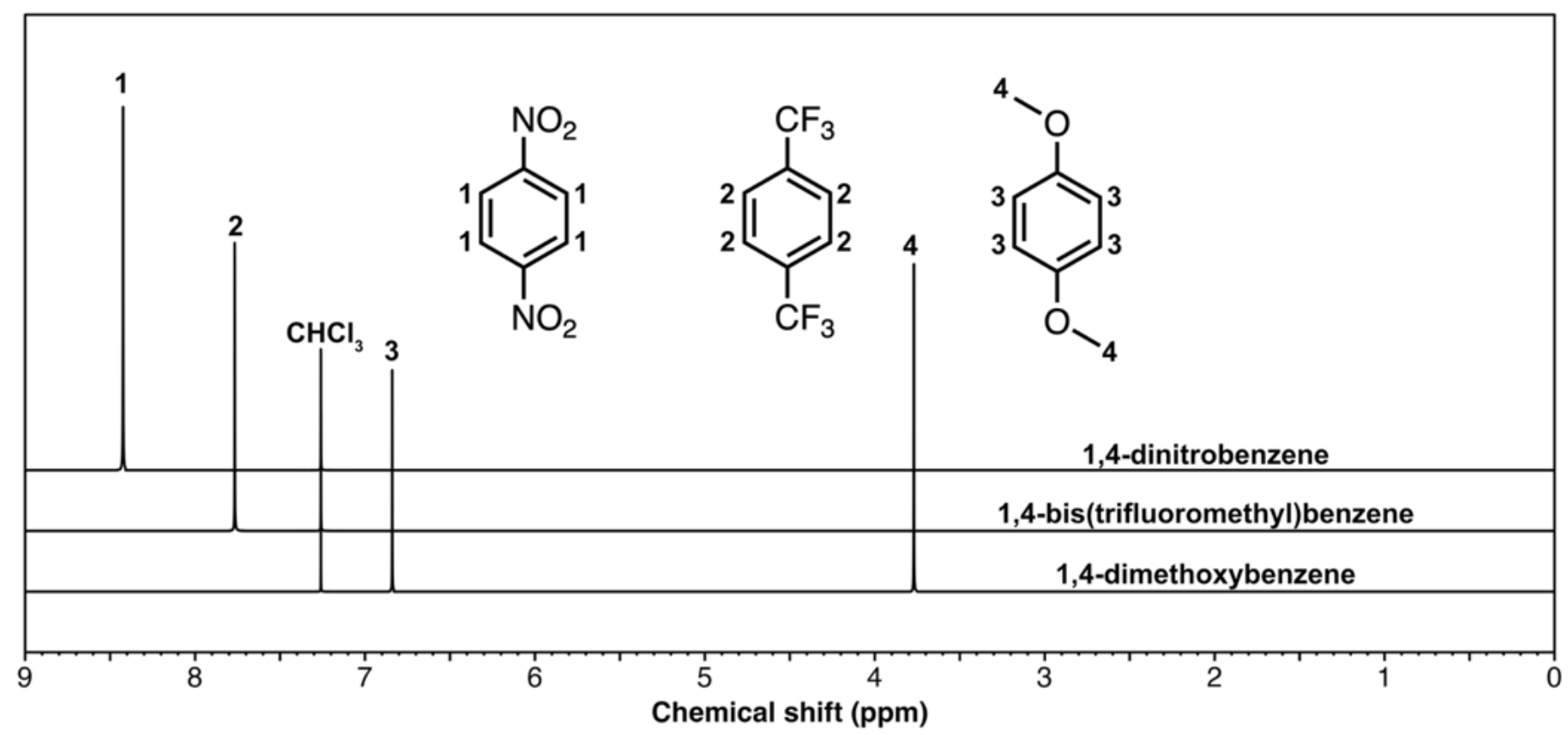

Figure S5. Annotated ^{1}H NMR spectra ($CDCl_3$, 400 MHz) and chemical structures of the internal standards 1,4-dinitrobenzene, 1,4-bis(trifluoromethyl)benzene, and 1,4-dimethoxybenzene. The peaks at ~7.26 ppm correspond to the proton signals of residual nondeuterated chloroform ($CHCl_3$).

Section S7.5 – Chemical structures and ^{1}H NMR spectra of pristine polyesters

Figures S6, **S7**, and **S8** show the chemical structures and ^{1}H NMR spectra of the tested polyesters poly(3-hydroxybutyrate-co-3-hydroxyhexanoate) (PHBH), poly(butylene adipate-co-terephthalate) (PBAT), and poly(butylene sebacate-co-terephthalate) (PBSeT), respectively.

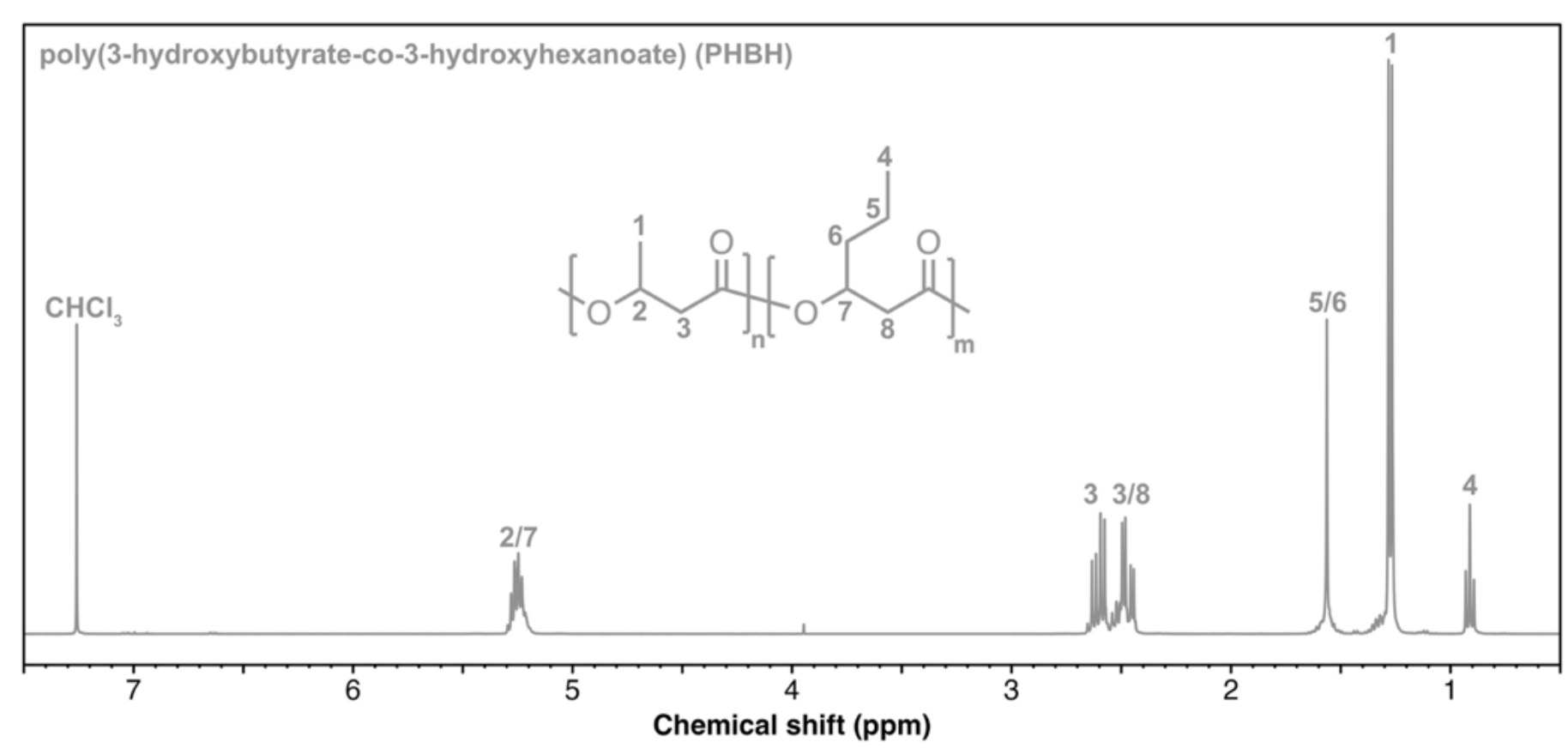

Figure S6. Annotated ^{1}H NMR spectrum ($CDCl_3$, 400 MHz) and chemical structure of used poly(3-hydroxybutyrate-co-3-hydroxyhexanoate) (PHBH). The depicted repeat units do not reflect the monomer fractions (see the Method section in the main manuscript). The peak at ~7.26 ppm corresponds to the proton signal of residual nondeuterated chloroform ($CHCl_3$).

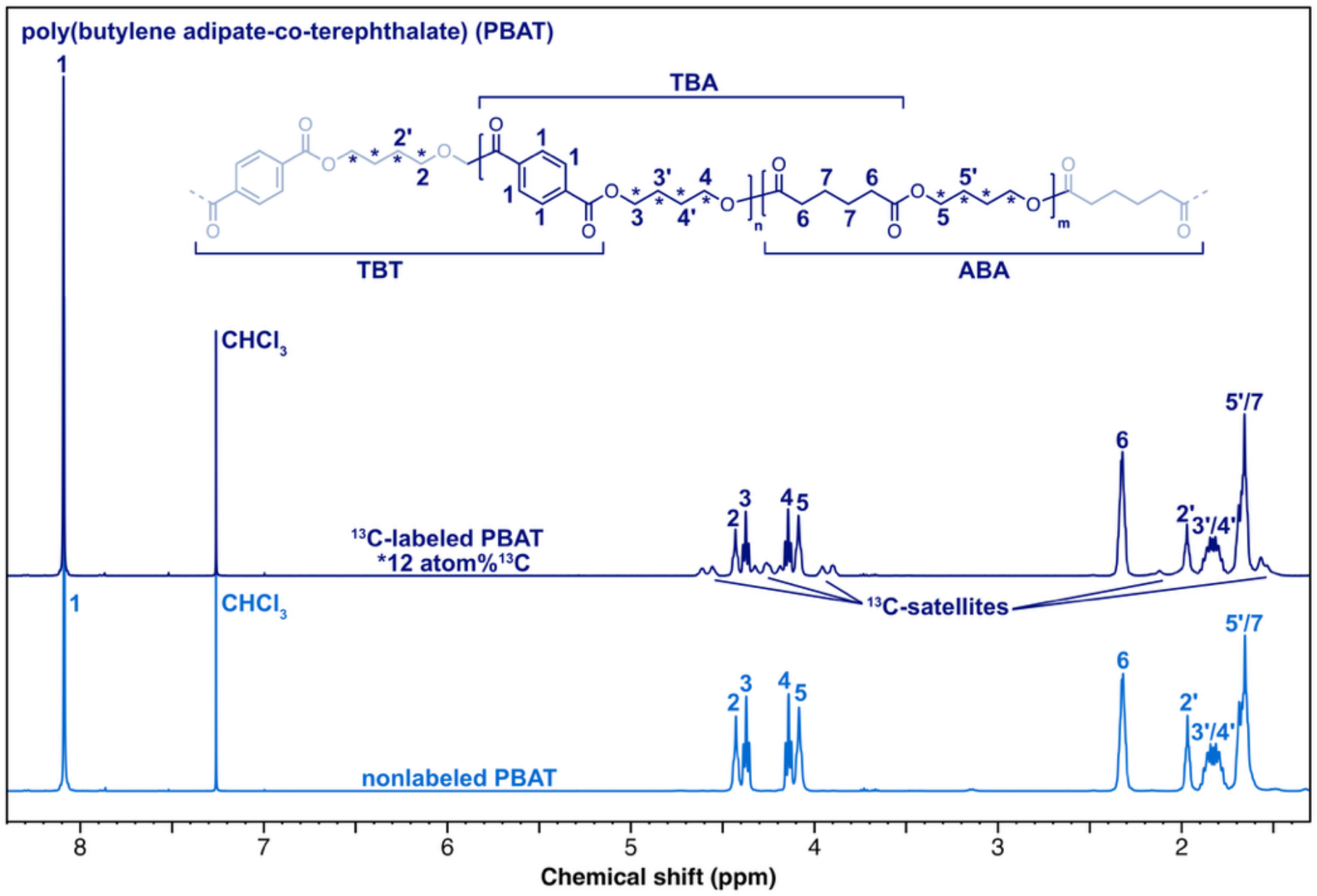

Figure S7. Annotated ^{1}H NMR spectra ($CDCl_3$, 400 MHz) and chemical structure of used ^{13}C-labeled and nonlabeled poly(butylene adipate-co-terephthalate) (PBAT). Peak splitting arises from variations in repeat unit sequences (i.e., terephthalate-butylene-terephthalate, TBT; terephthalate-butylene-adipate, TBA; adipate-butylene-adipate, ABA), therefore, additional repeat units (light shading) are included in the chemical structure to aid in signal assignment. The depicted repeat units in the chemical structure do not reflect the actual monomer fractions (see the Method section in the main manuscript). The peaks at ~7.26 ppm correspond to the proton signals of residual nondeuterated chloroform ($CHCl_3$).

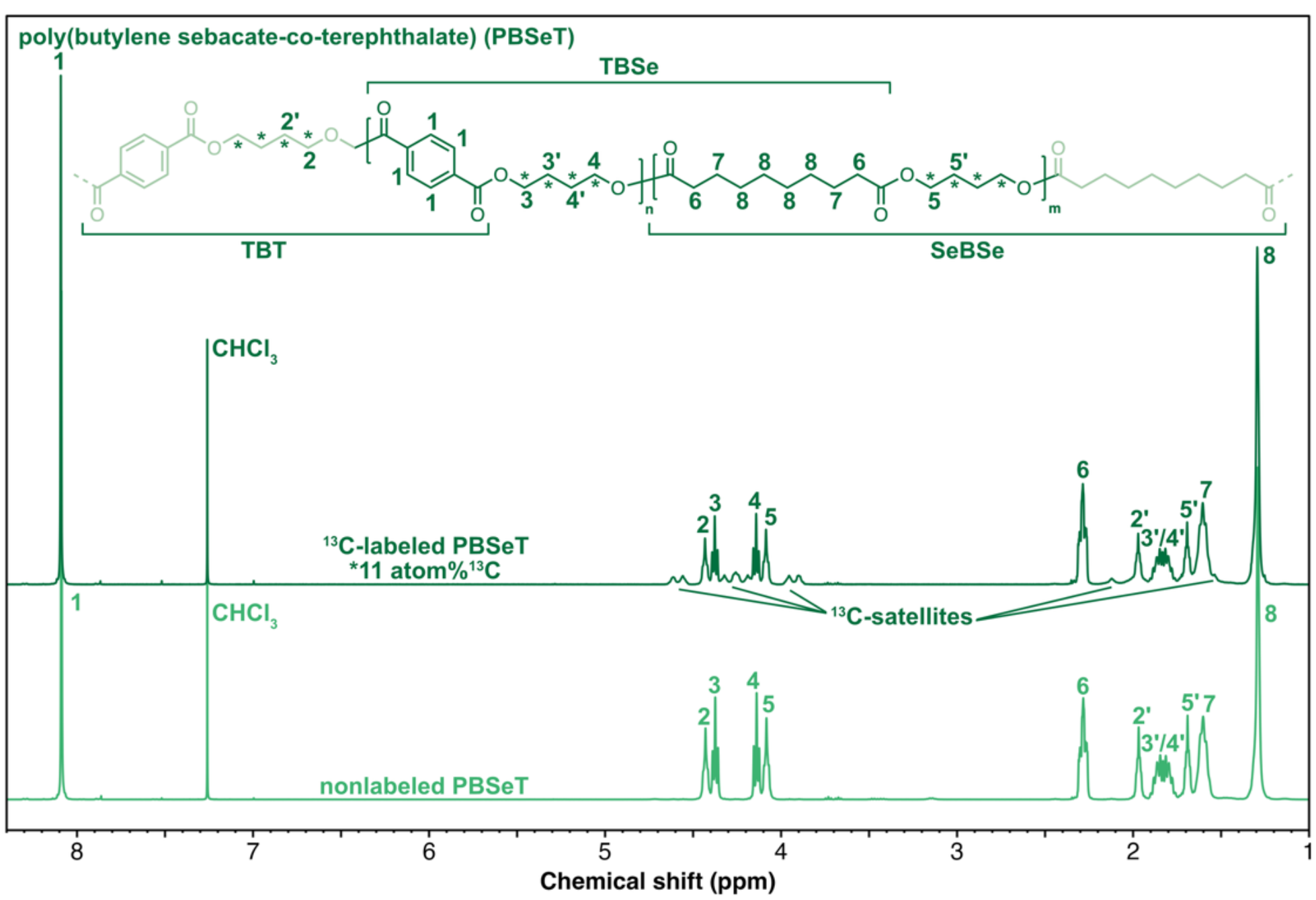

Figure S8. Annotated ^1H NMR spectra ($CDCl_3$, 400 MHz) and chemical structure of used ^{13}C-labeled and nonlabeled poly(butylene sebacate-co-terephthalate) (PBSeT). Peak splitting arises from variations in repeat unit sequences (i.e., terephthalate-butylene-terephthalate, TBT; terephthalate-butylene-sebacate, TBSe; sebacate-butylene-sebacate, SeBSe), therefore, additional repeat units (light shading) are included in the structure to aid in signal assignment. The depicted repeat units in the chemical structure do not reflect actual monomer fractions (see the Method section in the main manuscript). The peaks at ~7.26 ppm correspond to the proton signals of residual nondeuterated chloroform ($CHCl_3$).

Section S7.6 – ^1H NMR integrals for repeat unit quantification

This study assessed the biodegradation of copolyesters with monomeric compositions that may change during biodegradation. Consequently, the monomeric composition of each copolyester was quantified independently. **Figure S9** shows the proton peaks for the different monomeric units in the ^1H NMR spectra that were selected for the quantification. The peaks used for quantification were chosen to minimize interference from co-extracted background compost organic matter (COM). Details on peak selection are provided in **Section S8.2**.

The peaks corresponding to the aromatic protons of the terephthalate unit (T) at a chemical shift of ~8.09 ppm were used to quantify the butylene terephthalate (BT) repeat units of both PBAT and PBSeT. The peaks of the methylene protons of the butylene units adjacent to the ester oxygen (–CO–O–CH_2–) at a chemical shift of ~4.11 ppm were used to quantify the butylene adipate (BA) and butylene sebacate (BSe) repeat units of PBAT and PBSeT, respectively. The peaks of the α-methylene protons of 3-hydroxybutyrate (3HB) at ~2.62 ppm and of 3-hydroxyhexanoate (3HH) at ~2.48 ppm were used to quantify PHBH. The peaks at ~2.48 ppm represent the superposition of one of the two symmetric α-methylene protons of the 3HB unit with both α-methylene protons of the 3HH unit (**Figure S6**). To quantify 3HB, the integral of the peak at ~2.62 ppm, corresponding to one proton (half of the symmetric pair), was used. To quantify 3HH, the integral of the peak at ~2.48 ppm was corrected by subtracting the contribution from 3HB (i.e., the integral at ~2.62 ppm), now corresponding to the two α-methylene protons of 3HH. Note that for the ^{13}C-labeled

PBAT and PBSeT (butanediol-labeled), ^{13}C-satellite peaks were included in the BA and BSe integrations (**Figure S9**).

For all internal standards, the peaks corresponding to the four aromatic protons of the benzene rings were used for polyester quantification (**Figure S5**).

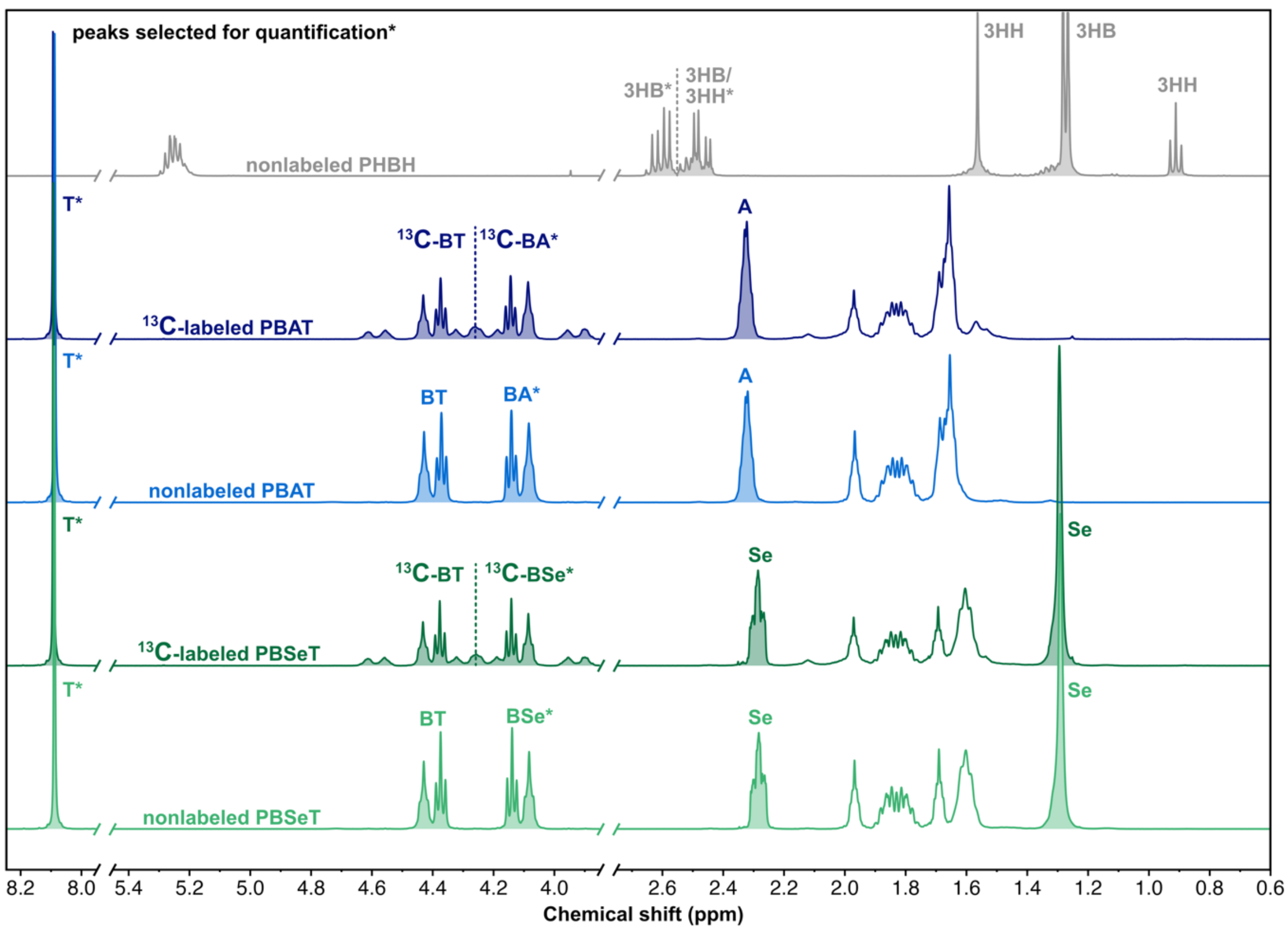

Figure S9. ^{1}H NMR spectra ($CDCl_3$, 400 MHz) of poly(3-hydroxybutyrate-co-3-hydroxyhexanoate) (PHBH; grey trace), ^{13}C-labeled and nonlabeled poly(butylene adipate-co-terephthalate) (PBAT; dark and light blue traces, respectively), and ^{13}C-labeled and nonlabeled poly(butylene sebacate-co-terephthalate) (PBSeT; dark and light green traces, respectively) with integrals suitable for repeat unit quantification (shaded) and integrals selected for quantification (*). These include integrals of terephthalate (i.e., T in PBAT and PBSeT), butylene terephthalate (i.e., BT in PBAT and PBSeT), butylene adipate (i.e., BA in PBAT), butylene sebacate (i.e., BSe in PBSeT), adipate (i.e., A in PBAT), sebacate (i.e., Se in PBSeT), 3-hydroxybutyrate (i.e., 3HB in PHBH), and 3-hydroxyhexanoate (i.e., 3HH in PHBH). Dashed lines indicate integration boundaries where these are not evident from the shaded regions.

Section S7.7 – Calculation of residual polyester mass and molar fractions

Calculation of repeat unit masses

Polyester amounts were expressed in carbon mass to allow for direct comparison with CRDS data (mass of mineralized $^{(13)}C$) and EA-IRMS data (mass of nonmineralized ^{13}C). First, the molar amounts of the individual repeat units (n_x, mmol) were calculated according to:

$$n_x = \frac{A_x}{H_x} \cdot \frac{n_{IS} \cdot H_{IS}}{A_{IS}} \qquad \textbf{Eq. S10}$$

where x denotes the repeat units BT, BA, BSe, 3HB, or 3HH; A_x and A_{IS} are the integrals of the selected ^1H NMR peak of x and the internal standard (arbitrary unit), respectively (see **Figure S9**); H_x and H_{IS} correspond to the number of protons that give rise to the peak; and n_{IS} the molar amount of internal standard added (mmol).

Carbon masses of the individual repeat units (m_x, mg C) were subsequently calculated by:

$$m_x = Mw_x \cdot n_x \qquad \textbf{Eq. S11}$$

where Mw_x denotes the molar carbon mass of the repeat unit (mg C $(mmol)^{-1}$), and n_x the molar amount (mmol) of the given repeat unit calculated by **Eq. S10**.

Chemical shifts of the peaks used for quantification, the corresponding number of protons, and both molar masses and molar carbon masses used for quantification are summarized in **Table S3**. Butanediol units make up 50 mol% of all monomeric units in the polyesters. To calculate the accurate molar mass of the BA/BSe and BT units of the ^{13}C-labeled polyesters, we accounted for the natural abundance (1.07 atom%) of ^{13}C in nonlabeled monomers and ascribed the excess number of ^{13}C atoms (mol%) to the BA/Se and BT repeating units based on their relative

abundance in the respective polyester (e.g., for PBAT, 46 mol% of the excess ^{13}C was ascribed to BT units and 54 mol% to BA units).

Table S3. Overview of parameters used for the calculation of residual polyester carbon masses.

**Polyester / Internal standard**	**Repeat unit**	**Chemical shift of ^{1}H NMR signal**	**Number of protons ^{1}H NMR signal, H_x**	**Molar mass of repeat unit (mg/mmol)**	**Molar carbon mass (mg C/mmol)**
Nonlabeled PBAT / *^{13}C-labeled PBAT**	BT	~8.09 ppm	4	220.22 / *221.42*	144.13 / *145.33*
	BA	~4.11 ppm	4	200.23 / *201.43*	120.11 / *121.30*
Nonlabeled PBSeT / *^{13}C-labeled PBSeT***	BT	~8.09 ppm	4	220.22 / *221.52*	144.13 / *145.43*
	BSe	~4.11 ppm	4	256.34 / *257.64*	168.15 / *169.45*
PHBH	HB	~2.62 ppm	1	86.09	48.04
	HH	~2.49 – 2.62 ppm	2	114.14	72.07
1,4-bis (trifluoromethyl)benzene	-	~7.77 ppm	4	214.11	96.09
1,4-dimethoxybenzene	-	~6.84 ppm	4	138.17	96.09
1,4-dinitrobenzene	-	~8.42 ppm	4	168.11	72.07

*labelling extent of ^{13}C: 12 atom%
**labelling extent of ^{13}C: 11 atom%

Compost background corrections

The used batches of industrial compost all contained Soxhlet co-extractable compost organic matter (COM). This COM can interfere with the accurate quantification of repeat units of (residual or spike-recovery added) polyesters. At low polyester concentrations, background

corrections for COM are necessary to ensure accurate quantification of polyesters (see **Section S8.4**).

In this work, background corrections were achieved by extracting blank compost samples that were incubated alongside the compost samples containing added polyesters. The blank compost ^{1}H NMR spectra were integrated over the chemical shift regions used for the calculation of polyester repeat units. Theoretical molar "equivalents" ($n_{x,\,blank}$; mmol) of the repeat units were calculated following **Eq. S10**. Background-corrected molar amounts ($n_{x,corr}$; mmol) for each repeat unit were then obtained by:

$$n_{x,\,corr} = m_{compost,\,sample} \cdot \left(\frac{n_{x,\,sample}}{m_{compost,\,sample}} - \frac{n_{x,\,blank}}{m_{compost,\,blank}} \right) \qquad \textbf{Eq. S12}$$

where $n_{x,\,sample}$ (mmol) is the molar amount of the respective repeat unit of the sample, $n_{x,\,blank}$ (mmol) the molar repeat unit equivalent of the blank compost sample (both with **Eq. S10)**, and $m_{compost,\,sample}$ and $m_{compost,\,blank}$ (g) are the extracted masses of freeze-dried compost of the sample and compost blank, respectively. Background-corrected carbon masses of the polyesters were then calculated by using $n_{x,corr}$ instead of n_x in **Eq. S11**.

This approach of background correction was preferred over simply subtracting the integrals of blank compost spectra from the integrals of polyester-containing compost spectra. By converting the integrals to molar equivalents and normalizing molar repeat unit amounts to the mass of compost extracted prior to subtraction, the chosen background correction approach accounts for both variations in the ^{1}H NMR response and in amounts of extracted compost.

Calculation of total residual polyester mass (both for spike-recovery experiments as well as for the analysis of incubated samples)

Total polyester carbon mass fractions were calculated by summing the masses of the repeat units calculated by **Eq. S11** (either background corrected, or non-background corrected) and normalizing to the initial polyester carbon mass added (mg C):

$$C_{\text{residual PBAT}}\,(\%) = 100 \cdot \frac{m_{BA} + m_{BT}}{m_{\text{PBAT added}}} \qquad \textbf{Eq. S13}$$

$$C_{\text{residual PBSeT}}\,(\%) = 100 \cdot \frac{m_{BSe} + m_{BT}}{m_{\text{PBAT added}}} \qquad \textbf{Eq. S14}$$

$$C_{\text{residual PHBH}}\,(\%) = 100 \cdot \frac{m_{3HB} + m_{3HH}}{m_{\text{PHBH added}}} \qquad \textbf{Eq. S15}$$

where $m_{\text{polyester added}}$ refers to the initial carbon masses of PBAT, PBSeT, or PHBH that were added to the sample (mg C).

Calculation of monomer fractions

The molar terephthalic acid to total diacid content (T content) of PBAT and PBSeT and the molar 3-hydroxyhexanoic acid to total 3-hydroxy acid content (3HH content) of PHBH were calculated as follows:

$$\text{T content (mol\%)} = 100 \cdot \frac{n_{BT}}{n_{BT} + n_{\text{BA or BSe}}} \qquad \textbf{Eq. S16}$$

$$\text{3HH content (mol\%)} = 100 \cdot \frac{n_{3HH}}{n_{3HH} + n_{3HB}} \qquad \textbf{Eq. S17}$$

where n_{BT}, n_{BA}, n_{BSe}, n_{3HH}, and n_{3HB} refer to the molar amounts of the BT, BA, BSe, 3HH, and 3HB repeat units obtained by **Eq. S10** or **Eq. S12**.

SECTION S8 – Quantification of residual polyester in compost by Soxhlet extraction coupled to quantitative ^{1}H NMR: method development

We have previously shown that Soxhlet extraction of polyesters coupled to quantitative ^{1}H NMR analysis enables accurate quantification of a large set of biodegradable polyesters (including PHBH, PBAT, and PBSeT) in soil.[2–4] In this section, we describe the steps taken to extend and validate the Soxhlet extraction and ^{1}H NMR method for polyesters in industrial compost samples. These steps include 1) the assessment of interferences from co-extractable compost organic matter (COM), 2) the determination of the number of Soxhlet extraction cycles required for complete polyester recovery in spike-recovery experiments, and 3) the determination of the effect of methanol (MeOH) pre-extraction to remove COM on polyester recoveries in spike-recovery experiments. All experiments focusing on analytical method development were conducted using nonlabeled PBAT and PBSeT.

Section S8.1 – Soxhlet extraction procedure

Compost was first freeze dried under a vacuum at 0.01 mbar for ~48 hours prior to extraction. Freeze-dried compost was either directly extracted or stored in a desiccator until being extracted. For each extraction, 11 grams of the freeze-dried compost, either with no polyester added or with varying amounts of PBAT or PBSeT added (described in detail in the individual sections), was transferred to cellulose extraction thimbles (VWR, 516−0252P). Thimbles were capped with a piece of polypropylene mesh (PROPYLTEX 05−150/34, Sefar S, mesh size 150 µm) to prevent compost particles from floating out of the extraction thimbles during the extractions.

All Soxhlet extractions were performed using a UniversalExtractor E-800 (Büchi, Switzerland), which allows to set the number of extraction cycles. One extraction cycle was

defined as the complete filling and subsequent draining of the extraction chamber containing the extraction thimble. Extractions were either conducted with a 9:1 $CHCl_3$:MeOH (vol:vol) mixture to directly extract the polyesters from the compost or involved a MeOH pre-extraction step to extract COM prior to using the 9:1 $CHCl_3$:MeOH (vol:vol) mixture to extract the polyesters. MeOH is a polar solvent that has been shown to selectively remove organic matter from soil matrices, thereby reducing spectral interference in ^{1}H NMR and enabling more accurate polyester quantification.[4]

All pre-extractions and extractions consisted of 10 extraction cycles, unless specified otherwise. For analyses including a MeOH pre-extraction, the MeOH of the pre-extractions was not analyzed and directly discarded. After completion of the polyester extraction cycles, the volume of the 9:1 $CHCl_3$:MeOH (vol:vol) mixture was reduced to a few milliliters by evaporation. The extract was then left overnight to fully dry at room temperature. Sample reconstitution, collection of ^{1}H NMR spectra, and processing of spectra was carried out as described in **Sections S7.1**, **S7.2**, and **S7.3**.

Section S8.2 – Evaluation of background compost organic matter extracts and polyester concentrations

We first determined the intensity and variability of the background signals in extracts of blank compost samples. These signals had contributions not only from extractable compost background organic matter (COM) but, in many cases, also from traces of biodegradable polyesters that were present in the obtained industrial compost. These traces of biodegradable polyester could interfere with accurate quantification of the added biodegradable polyesters, particularly when they were added in only low amounts in spike-recovery experiments.

We extracted a total of 24 blank compost samples (each of 11.0 ± 0.0 grams dry compost weight). Half (i.e., 12) of these samples first underwent a MeOH pre-extraction step prior to the

$CHCl_3$:MeOH extraction step. The other 12 samples were directly extracted with $CHCl_3$:MeOH. **Figure S10** shows the superimposed ^{1}H NMR spectra of the extracted blank compost samples over the chemical shift ranges chosen for polyester quantification. **Figure S11** shows the respective polyester mass 'equivalents' per gram of dry compost, determined by integration of ^{1}H NMR signals over the chemical shift ranges used for polyester quantification (**Section S7.6**).

All spectra of blank compost extracts in **Figure S10** exhibited elevated signals. At the chemical shifts corresponding to the repeat units T (~8.09 ppm) and BT (~4.41 ppm), peaks characteristic of these repeat units were observed in the blank compost extracts, indicating that the compost contained traces of nonbiodegraded BT unit-containing polyesters. At the chemical shifts of butylene C_1 protons in butylene adipate (BA) and butylene sebacate (BSe, ~4.11 ppm), none of the characteristic peaks of these units were discernible in the spectra, suggesting that these repeat units were either absent or masked by signals from COM. At lower chemical shifts, interference from COM was so pronounced that no characteristic polyester peaks were detectable. Overall, the MeOH pre-extraction step appeared to have broadly decreased interference by co-extractable COM, particularly at chemical shifts below ~2.2 ppm.

This finding is supported by **Figure S11**, which shows that the corresponding repeat unit mass equivalents of the extracted compost were lower when the $CHCl_3$:MeOH extraction step was preceded by the MeOH pre-extraction. **Figure S11** shows that the MeOH pre-extraction was particularly effective in removing interferences in the chemical shift regions used for BA, A, BSe, and Se repeat unit quantification. Including a compost MeOH pre-extraction step therefore was expected to increase quantification accuracies of polyesters, particularly when the polyesters were present at low concentrations.

Figure S10 and **S11** show that there were severe interference from co-extracted COM at low chemical shifts (i.e., below ~2.4 ppm). This finding implies that selecting downfield repeat unit peaks, which were less susceptible to COM interference, is preferable for polyester quantification. Herein, we therefore used the T signal (chemical shift of ~8.09 ppm) for the quantification of BT units in PBAT and PBSeT. For the quantification of BA and BSe units, we selected the peaks at chemical shifts of ~4.11 ppm, because the A peaks at ~2.32 ppm and the Se peaks at ~2.28 and ~1.29 ppm consistently showed higher interference from COM, both for samples with and without a MeOH pre-extraction step. For PHBH, the chemical shift regions below ~2 ppm showed strong interference from co-extracted COM. Consequently, the 3HB unit was quantified using the proton peaks at ~2.55 ppm, and the 3HH unit using the peaks at ~2.49 ppm.

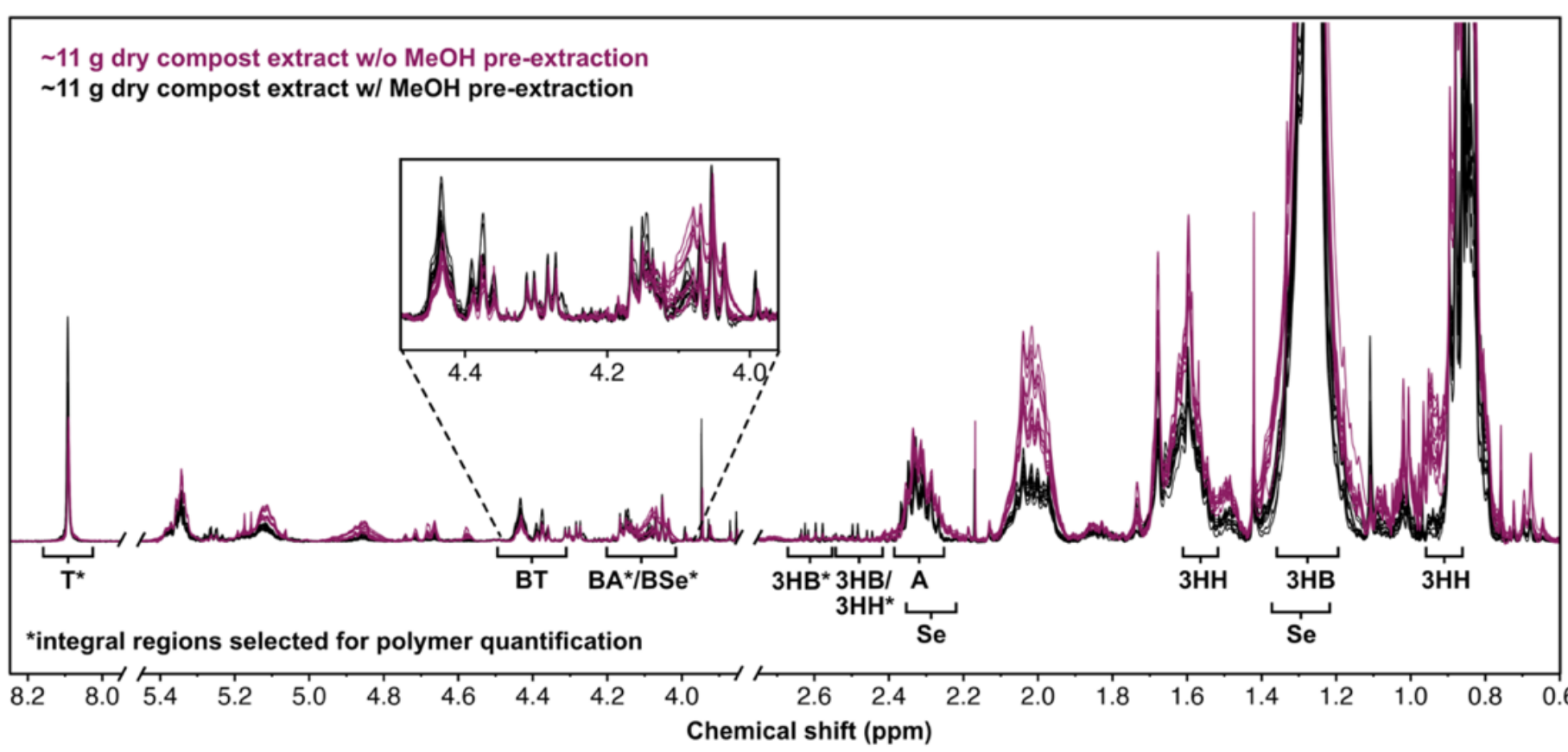

Figure S10. ^{1}H NMR spectra ($CDCl_3$, 400 MHz) from Soxhlet extractions of 24 blank compost samples. Half of these samples were directly extracted with a 9:1 $CHCl_3$:MeOH (vol:vol) mixture (purple trace; labeled 'w/o MeOH pre-extraction'). The other half of the samples underwent a methanol (MeOH) pre-extraction prior to the main extraction with the 9:1 $CHCl_3$:MeOH (vol:vol) mixture (black trace; labeled 'w/ MeOH pre-extraction'). The abscissa specify the chemical shift regions corresponding to the proton signals of the repeat units terephthalate (T in PBAT and PBSeT) at ~8.09 ppm, butylene terephthalate (BT in PBAT and PBSeT) at ~4.41 ppm, butylene adipate (BA in PBAT) and butylene sebacate (BSe in PBSeT) at ~4.11 ppm, adipate (A in PBAT) at ~2.32 ppm, sebacate (Se in PBSeT) at ~2.28 and at ~1.29 ppm, 3-hydroxybutyrate (3HB in PHBH) at ~2.55 and at ~1.27 ppm, and 3-hydroxyhexanoate (3HH in PHBH) at ~2.49, at ~1.56, and at 0.91 ppm. The respective polyester mass equivalents per mass of dry compost corresponding to these integrals are given in **Figure S11**.

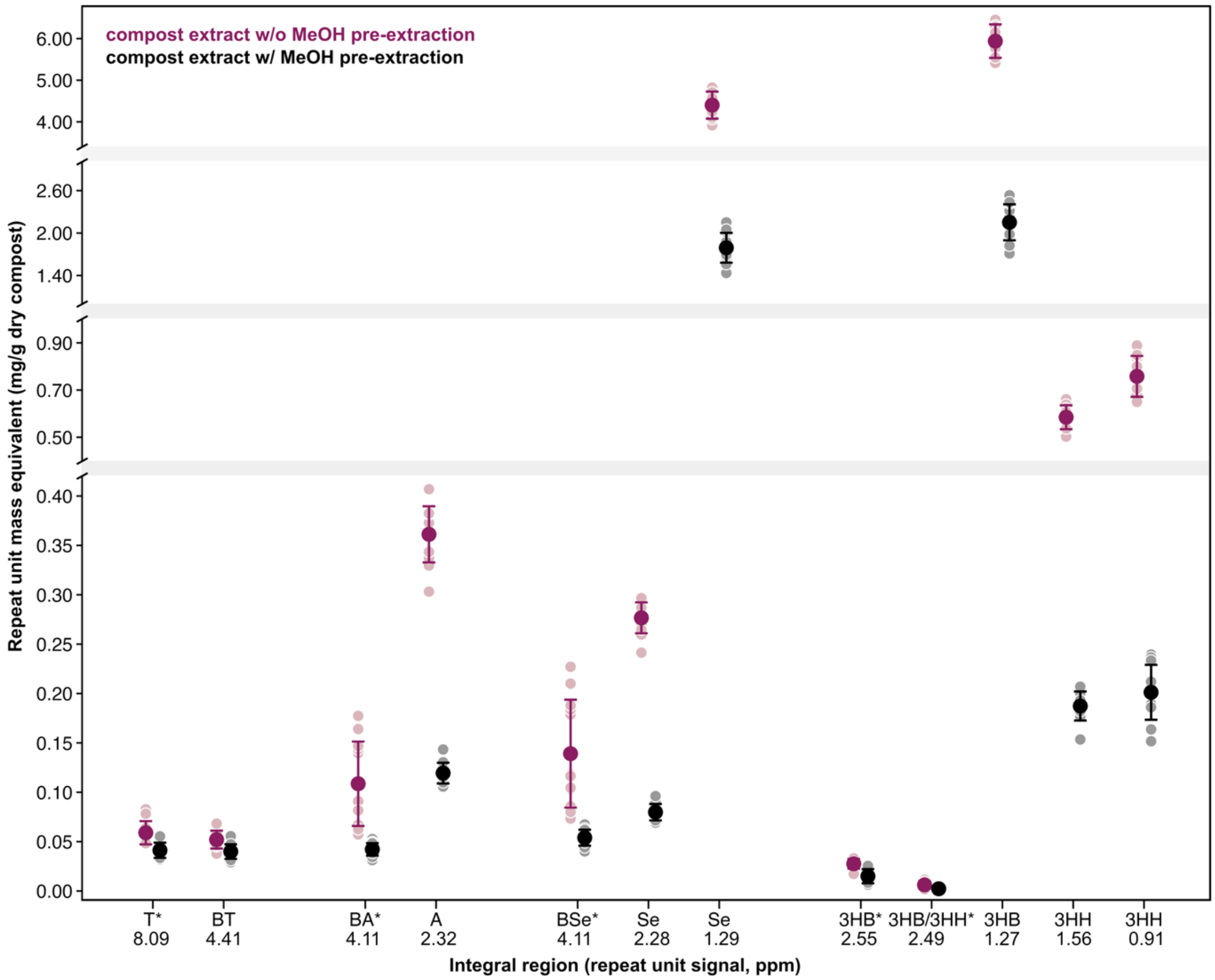

Figure S11. Mass equivalents of polyester repeat units obtained from Soxhlet extractions of 24 blank compost samples, of which 12 samples were directly extracted with a 9:1 $CHCl_3$:MeOH (v:v) mixture (purple trace; labeled "w/o MeOH pre-extraction"), and 12 underwent a methanol (MeOH) pre-extraction prior to the main extraction with the 9:1 $CHCl_3$:MeOH (v:v) mixture (black trace; labeled "w/ MeOH preextraction"). Repeat units include terephthalate (T), butylene terephthalate (BT), butylene adipate (BA), adipate (A), butylene sebacate (BSe), sebacate (Se), 3-hydroxybutyrate (3HB), and 3-hydroxyhexanoate (3HH). Dark data points represent the average repeat unit mass equivalents, and error bars indicate the corresponding standard deviations. Individual data points of the 12 samples are shown in lighter shades. Corresponding ^{1}H NMR spectra are shown in **Figure S10**.

Section S8.3 – Effect of the number of soxhlet extraction cycles on polyester recoveries

We determined the number of extraction cycles needed for complete recovery of polyesters spiked to the blank compost samples. To this end, 10 mg of either PBAT or PBSeT was spiked into

11 g of freeze-dried blank compost, followed by extraction for 1, 2, 5, 10, 15, and 20 cycles. For each number of extraction cycles and both polyesters, duplicate extractions were run. Extractions were performed with 9:1 $CHCl_3$:MeOH (vol:vol) without a MeOH pre-extraction.

Figure S12 shows both uncorrected and background-corrected recoveries of the spiked PBAT and PBSeT. Recoveries largely increased with increasing number of extraction cycles up to a total of 5 extraction cycles. Increasing the number of extraction cycles beyond 10 did not substantially improve extraction efficiencies. Without background correction, recoveries consistently exceeded 100% at five and more extraction cycles (closed symbols in **Figure S12**). Background correction (i.e., subtraction of 'equivalent polyester amounts' in extracts of blank compost samples, see **Section S7.7**) lowered recoveries to close to 100% (open symbols in **Figure S12**). The data showed that a total of 10 extraction cycles were sufficient to achieve complete recovery of spiked polyesters.

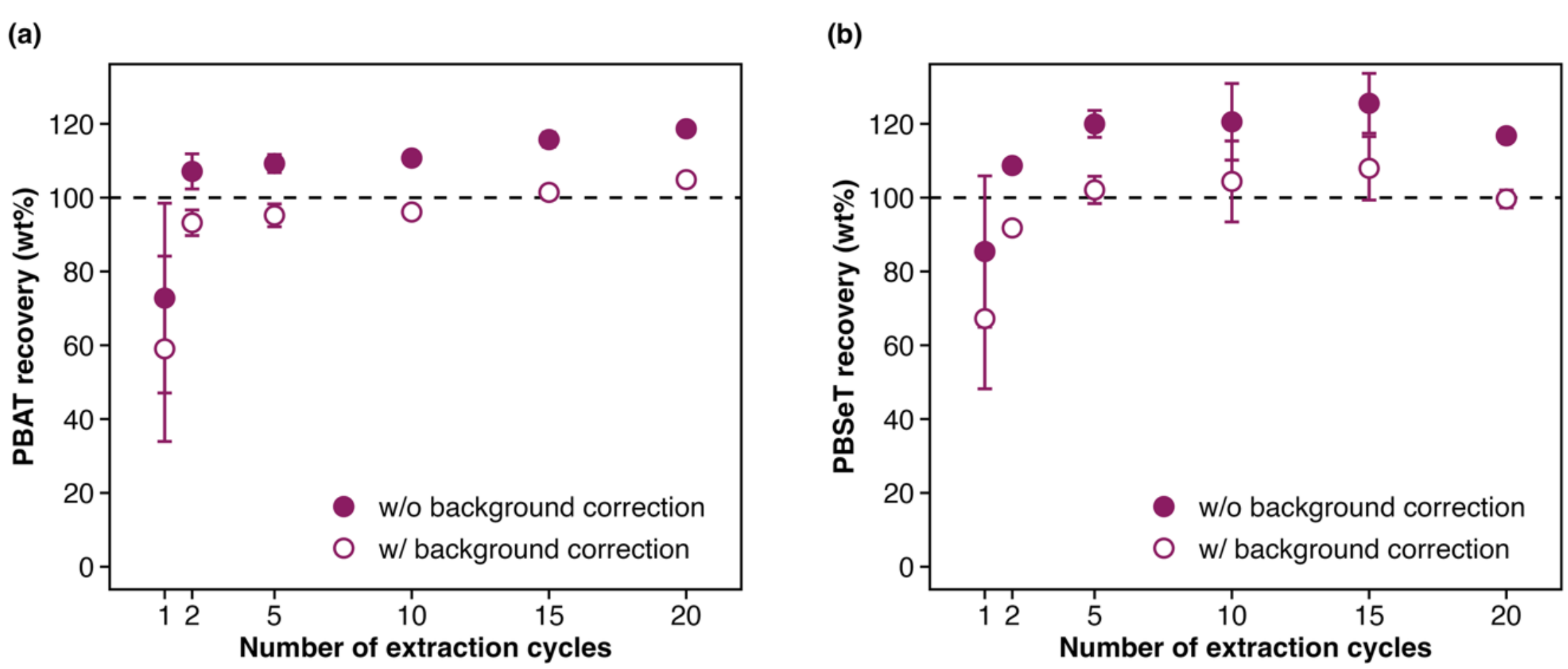

Figure S12. Percent mass recoveries of **(a)** poly(butylene adipate-co-terephthalate) (PBAT), and **(b)** poly(butylene sebacate-co-terephthalate) (PBSeT) as a function of number of consecutive Soxhlet $CHCl_3$:MeOH extraction cycles. Spike-recovery experiments were performed in duplicate for each number of extraction cycles tested with 10 mg polyester added into 11 g freeze-dried compost. Samples were extracted with 9:1 $CHCl_3$:MeOH (vol:vol) without a MeOH pre-extraction. Data points represent mean recoveries of duplicate samples without background correction (filled circles) and with background correction (open circles). Error bars show the absolute ranges between the duplicate samples.

Section S8.4 – Spike-recovery experiments

We conducted spike-recovery experiments in which varying amounts of PBAT or PBSeT were added to 11 g of compost (each in triplicates), followed by determining polyester recoveries by CH_3Cl:MeOH extraction both with and without MeOH pre-extraction. Additionally, for each added polyester mass, recoveries were determined with and without background compost correction following the procedure described above. Results are presented in **Figure S13**.

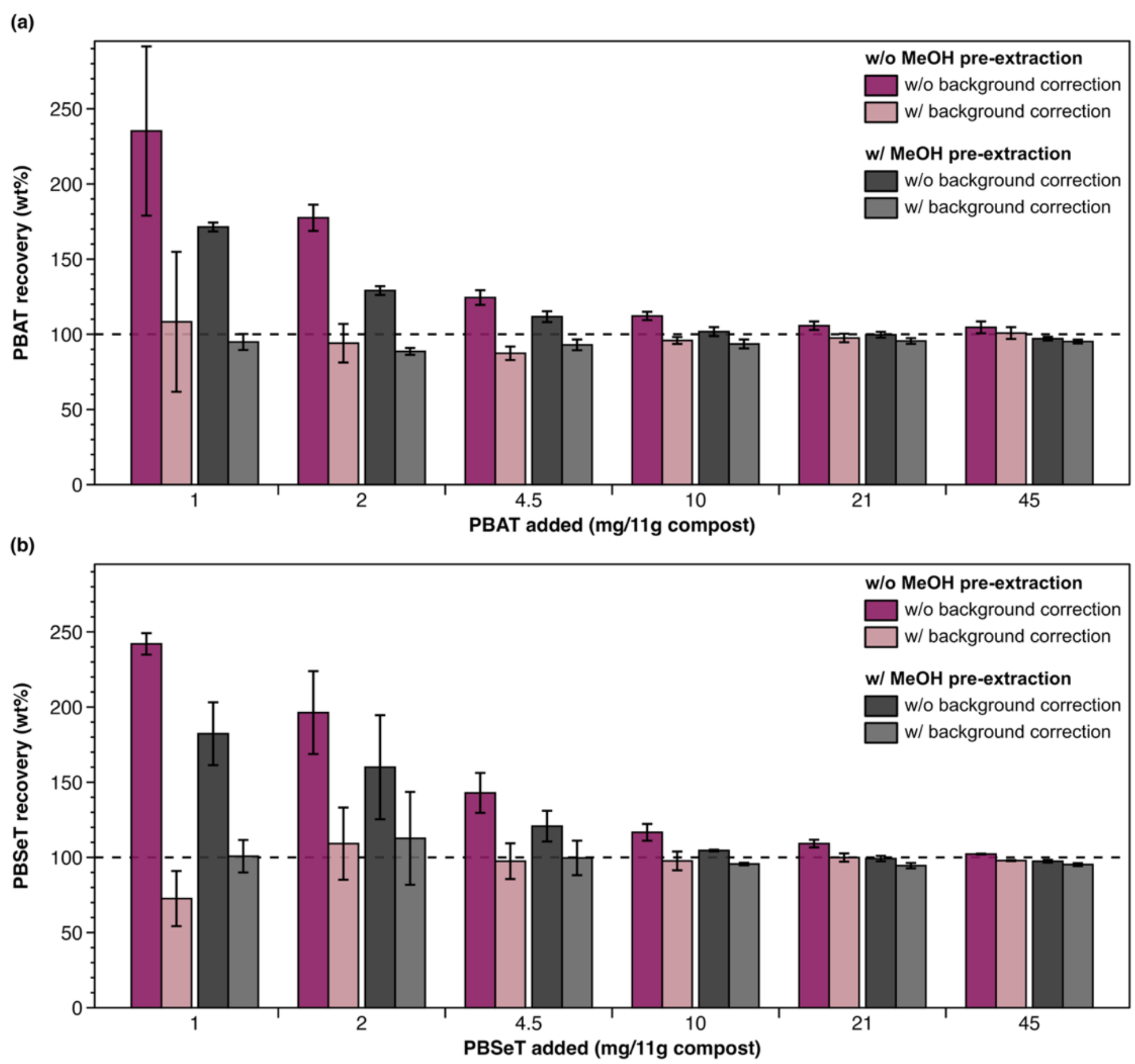

Figure S13. Average percent mass recoveries of polyesters **(a)** poly(butylene adipate-co-terephthalate) (PBAT) and **(b)** poly(butylene sebacate-co-terephthalate) (PBSeT) used in spike-recovery experiments at varying polyester amounts spiked into the compost samples. Bars represent mean recoveries of triplicates, and error bars denote standard deviations. Results are shown for direct polyester extraction without a methanol (MeOH) pre-extraction (purple shades), and for samples subjected to a MeOH pre-extraction prior to polyester extraction (grey shades). Lighter-shaded bars indicate data corrected for average polyester mass equivalents determined from blank compost extractions (i.e., background correction; see **Section S7.7**).

At the lower spiked masses of polyesters (i.e., 1, 2 and 4.5 mg per 11 g dry compost), extracts without MeOH pre-extraction and background correction showed apparent PBAT and PBSeT mass recoveries above 100%, implying substantial over-quantification by interferences from co-extracted COM. Background correction corrected for this effect but led to incomplete polyester recoveries for some samples. This apparent under-quantification was likely due to the high variability in the signals in blank compost extracts (due to variations in co-extracted COM but possibly also co-extracted polyesters present in the blank compost samples) and thus the 'average' blank compost signals exceeding the actual background signal in a given sample to which low polyester masses were spiked. As expected, including a MeOH pre-extraction decreased over-quantification. The combination of MeOH pre-extraction and, additionally, background correction yielded recoveries closest to 100%. Nonetheless, at low added polyester masses of 1 and 2 mg, recoveries were variable around 100% even with corrections, suggesting that sample-to-sample variations in the amounts of co-extracted COM could not be fully addressed by the mean of extracted blank replicate samples also for MeOH pre-extracted samples.

At higher added polyester amounts (i.e., 10, 21, and 45 mg per 11 g dry compost), no major differences were observed between extractions with or without MeOH pre-extraction, and with or without background correction. Thus, when polyester concentrations in a compost sample are expected to fall into this concentration range, the additional steps can be omitted from the analyses.

In summary, for PBAT and PBSeT, concentrations of roughly >1 mg (g dry compost)$^{-1}$, direct extraction with 9:1 $CHCl_3$:MeOH (vol:vol) are sufficient for reliable quantification of polyester. For polyester concentrations below approximately 1 mg (g dry compost)$^{-1}$, both MeOH pre-extraction and background correction are advised for accurate polyester quantification.

Section S8.5 – Signal-to-noise and limits of detection and quantification

The spike-recovery experiments were not designed to determine the limits of detection (LOD) and quantification (LOQ) of the extraction method. Accordingly, LOD and LOQ values are not reported herein. Notably, LOD and LOQ likely are strongly dependent on the type of industrial compost used, and are expected to largely depend on the variations in background levels of compostable polyesters they contain (i.e., any residual particulate biodegradable polyesters will result in elevated, specific background signals that may substantially increase LOD and LOQ of the same (or chemically similar) polyesters) and the variations in extractable compost organic matter. That is, reliable LOD/LOQ determination would need to be performed for each given compost. The experimental procedure to determine LOD and LOQ for Soxhlet extraction and ^{1}H NMR quantification has been published for soils.[4]

While we do not report LOD and LOQ, we confirmed that the signals obtained by extracting polyester were well-above the signal-to-noise ratio (S/N). To this end, we determined the S/N of polyester peaks in ^{1}H NMR spectra using the "SNR Calculation" function in MestReNova software. At the lowest added polyester mass in the spike-recovery experiments (i.e., 1 mg polyester per 11 g dry compost, followed by reconstitution of the dried extract in 3 mL internal standard $CDCl_3$ solution; **Section S8.4**), polyester peaks exhibited S/N ratios of > 500 for the T peak (~8.09 ppm) and > 100 for BA and BSe peaks (~4.11 ppm), for samples both with and without MeOH pre-extraction. These values are well above commonly accepted S/N thresholds of 3 for LOD and 10 for LOQ.

Likewise, all reported residual polyester concentrations for both experimental sets 1 and 2 (**Section S9**) corresponded to NMR signals much higher than 10 times the S/N values of background spectra. We did not use S/N values to estimate LOD/LOQ values from spike-recovery

responses, given that spike-recovery experiments and experiments were conducted at concentrations above LOD/LOQ. In general, LOD and LOQ are expected to be strongly-compost dependent because of variable compost organic matter co-extraction and potentially present residual amounts of compostable plastics (that would result in specific NMR features that could interfere with quantification of a polyester added, for instance for biodegradation studies). Therefore, we recommend performing LOD/LOQ specific spike-recovery experiments in future studies in which polyester concentrations approach S/N values.

SECTION S9 – Quantification of residual polyester in compost by soxhlet extraction coupled to quantitative ^{1}H NMR: incubation experiments

This section describes the application of the developed Soxhlet extraction method to the compost incubation experiments and highlights modifications made to the extraction procedure as compared to the method development described in **Section S8**.

Section S9.1 – Set 1 (^{13}C-labeled polyesters)

Soxhlet extractions for set 1 were carried out using a manual heating setup (heating apparatus: R306S, Behr LaborTechnik; extractor chamber: EZ30H, Behr LaborTechnik). The Behr setup was selected as it allows for higher sample throughput. At the same time, it was limited to smaller extraction thimbles (and thus smaller compost masses) as compared to the UniversalExtractor E-800 that was used for method development (**Section S8**).

To verify complete polyester extraction with the new setup and sample preparation, we conducted polyester-compost spike-recovery experiments prior to starting the incubation extractions. Three concentrations of 5, 25, and 50 mg of PBAT or PBSeT spiked to 9 g freeze-dried compost were tested in duplicate, with and without MeOH pre-extraction and with and without background correction. The results are shown in **Figure S14**. While confirming the

findings in **Section S8.4**, the results show that background correction of the MeOH pre-extracted samples resulted in incomplete PBAT and PBSeT recoveries of approximately 5-15%. The ^1H NMR spectra revealed that no polyester was present in the MeOH extracts and there was no evidence for methanolysis of the polyesters.

We therefore performed an additional extraction experiment in which PBAT and PBSeT (5, 25, and 50 mg; in duplicates) were added directly to cellulose extraction thimbles without compost. Following the extraction procedure above, extraction efficiencies of 97 ± 1 mol% BT and 96 ± 1 mol% BA for PBAT, and of 94 ± 1 mol% BT and 90 ± 0 mol% BSe for PBSeT were obtained. When applying these extraction efficiencies to the above MeOH pre-extracted samples with background correction, average polyester recoveries increased to approximately 100% (**Figure S14**).

The compost samples (and thus also the extracts) of experimental set 1 had considerably lower mass loadings than of set 2 (i.e., starting concentration of ~5 mg polyester (g dry compost)$^{-1}$, corresponding to ~45 mg per 9 g of compost in set 1, as compared to a starting concentration of ~100 mg polyester (g dry compost)$^{-1}$ in set 2). For set 1 samples, we therefore included a MeOH pre-extraction to remove co-extractable COM, performed the compost background correction, and additionally adjusted for extraction efficiency (mass loss of polyesters from empty thimbles), as detailed above. Given that the Behr extraction setup worked with smaller thimbles, ~9 g of freeze-dried sample was extracted in duplicate from each incubation bottle. We considered that the total compost mass of approximately 18 g being extracted was sufficient to ensure that it was representative of the total compost sample (63 ± 3 g per incubation bottle).

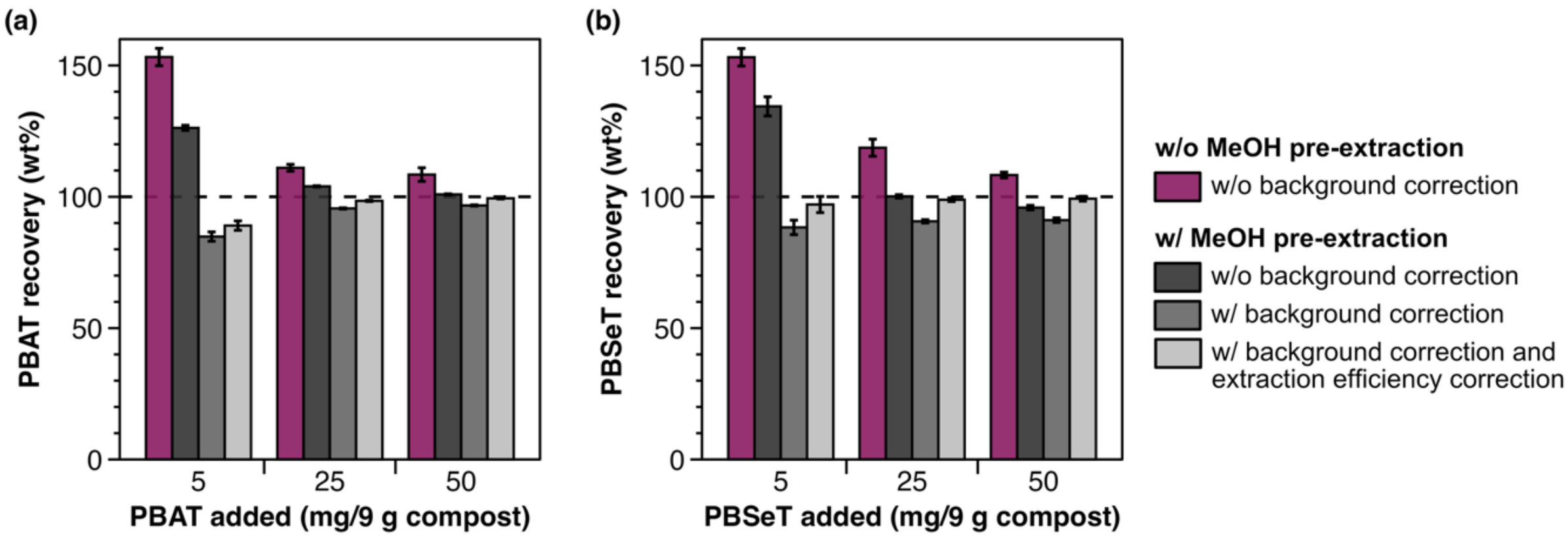

Figure S14. Average percent mass recoveries of polyester-compost spike-recovery experiments with varying added masses of **(a)** poly(butylene adipate-co-terephthalate) (PBAT) and **(b)** poly(butylene sebacate-co-terephthalate) (PBSeT). Bars represent mean recoveries of duplicates, and error bars correspond to the absolute range between the duplicates. Results are shown for direct polyester extractions without a methanol (MeOH) pre-extraction (purple shades) and for samples subjected to a MeOH pre-extraction prior to polyester extraction (grey shades). Lighter-shaded bars indicate data corrected for average polyester mass equivalents determined from blank compost extractions (i.e., background correction; see **Section S7.7**), and data corrected for extraction efficiency (see **Section S9.1** for details).

Section S9.2 – Set 2 (nonlabeled polyesters)

Similar to the procedure described in **Section S8.1**, all extractions for set 2 were conducted using a UniversalExtractor E-800 (Büchi, Switzerland). For this experiment, however, larger aliquots of ~15-20 g of freeze-dried compost sample (~25% of the total sample mass) were extracted to ensure that the taken subsample was representative of the incubated compost sample. Given the high mass loadings in set 2 (starting concentration of ~100 mg polyester (g dry compost)$^{-1}$), direct extraction with 9:1 $CHCl_3$:MeOH (vol:vol) was applied, as supported by the results in **Section S8.4**.

Initially, 10 extraction cycles were performed (see **Section S8.3**). Due to the substantially higher mass loadings compared with **Section S8.4**, two additional sets of 10 extraction cycles were performed to assess whether the polyester extractions were exhaustive. From the extract of the first 10 extraction cycles, an aliquot of ~3 mL was removed, dried, and prepared for ^{1}H NMR analysis

following **Section S7.1**. This step was necessary because polyester masses in the extracts were too high to allow reconstitution of the entire extracted polyester mass in a few milliliters of $CDCl_3$. The extracts of the second and third sets of 10xft extract cycles were analyzed as usual following **Section S7.1**. The second set of 10 extraction cycles contained additional polyester in the extract, indicating that the first 10 cycles were insufficient for complete extraction of the polyester at the high mass loadings. However, the third set of 10 extraction cycles did not extract significant amounts of polyesters, indicating that a total of 20 extraction cycles was sufficient to ensure complete polyester extraction. The polyester mass recoveries reported in the manuscript thus represent the combined extracted masses from the first 20 extraction cycles. Due to the high polymer mass loadings, we did not perform a compost background correction.

SECTION S10 – Mineralization rates

Mineralization rates of set 1 (i.e., ^{13}C-labeled PBAT, ^{13}C-labeled PBSeT, and ^{13}C-labeled cellulose) and set 2 (nonlabeled PBAT, nonlabeled PBSeT, and nonlabeled PHBH) are shown in **Figure S15**. Mineralization extents presented in **Figure 1** in the main manuscript were obtained by time-integration of these rates.

Initial mineralization rates were low (but exhibited a small peak during the first few hours of incubation, likely corresponding to microbial utilization of monomers and low-molecular-weight oligomers remaining after synthesis that readily diffused out of the polyesters), followed by the second phase in which mineralization rates peaked, and finally the third phase in which mineralization rates decreased to very small (but non-zero) final values.

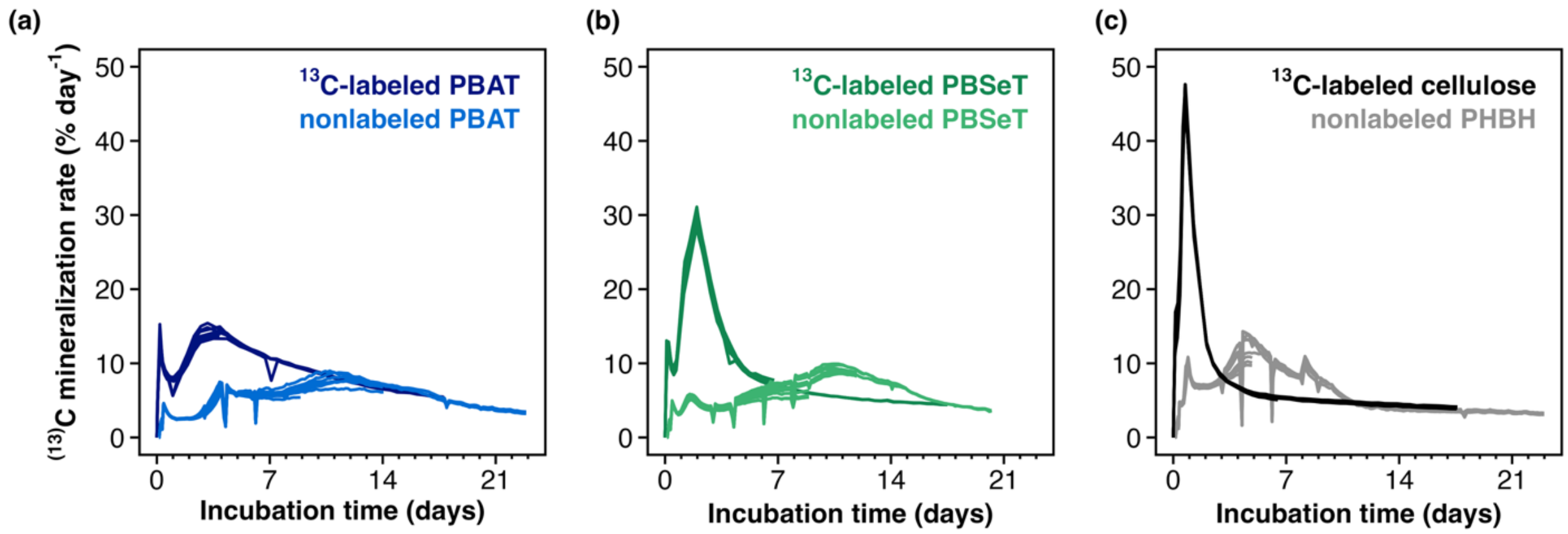

Figure S15. Mineralization rates, expressed as percent of added $^{(13)}$C per day, for ^{13}C-labeled and nonlabeled polyesters: **(a)** poly(butylene adipate-co-terephthalate) (PBAT), **(b)** poly(butylene sebacate-co-terephthalate) (PBSeT), and **(c)** ^{13}C-labeled cellulose and nonlabeled poly(3-hydroxybutyrate-co-3-hydroxyhexanoate) (PHBH).

SECTION S11 – Blank compost CO_2 formation

Figure S16 shows the CO_2 formation rates of the blank compost incubation bottles (no added polymers) of set 1 (incubations with ^{13}C-labeled substrates) and set 2 (incubation with nonlabeled polyesters). The two experiments were conducted with two different batches of compost (see **Section S3**).

Figure S16 shows that the background mineralization rates of the two batches were comparable, suggesting comparable microbial activities between the compost batches and thus supporting that mineralization dynamics of polyesters between set 1 and set 2 can be compared.

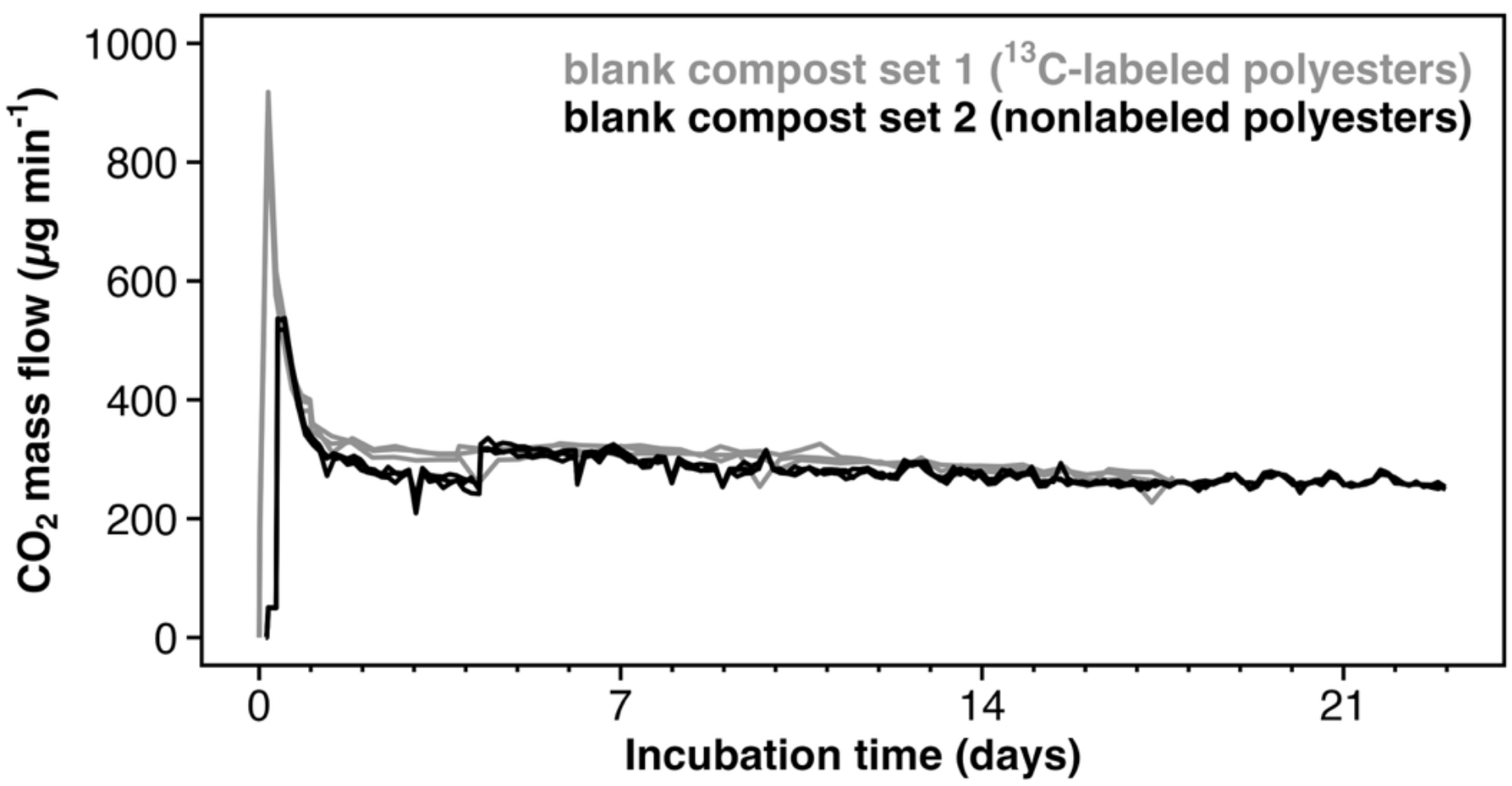

Figure S16. Background carbon dioxide (CO_2) mass flows from blank compost samples (no polymer added), expressed in micrograms CO_2 per minute. Data are shown for set 1 (grey trace, compost used for incubations with ^{13}C-labeled substrates) and set 2 (black trace, used for incubations with nonlabeled polyesters). The experiments were conducted with two different batches of compost (see **Section S3**). The laboratory air background (approximately 450–500 ppm CO_2, corresponding to approximately 81–90 µg CO_2 min^{-1}) was subtracted from these data.

SECTION S12 – Overview of biodegradation data

Table S4 summarizes values obtained per incubation bottle and treatment averages for $^{(13)}C_{mineralized}$, $^{13}C_{nonmineralized}$, $^{13}C_{mass\ balance}$, $^{(13)}C_{residual\ polyester}$, $^{(13)}C_{biomass}$, and calculated carbon use efficiency (CUE) values. Further information is provided in the table footnotes.

Table S4. Biodegradation data from set 1 (incubations with ^{13}C-labeled substrates) and set 2 (incubations with nonlabeled polyesters), shown for individual incubation bottle replicates and for treatment averages in bold. When applicable, absolute ranges between technical duplicates are given in parentheses.

Substrate	Replicate bottle	Incubation time (days)	$^{(13)}C_{mineralized}$	$^{13}C_{nonmineralized}$	Mass balance on ^{13}C[a]	$^{(13C)}C_{residual\ polyester}$[b]	$^{(13)}C_{biomass}$ (estimate 1)[c]	$^{13}C_{biomass}$ (estimate 2)[d]	Estimated CUE[e]
			(% of added polyester $^{(13)}C$)						
^{13}C-labeled PBAT (set 1)	1	4	18.0	74.7 (± 0.2)	92.7	85.4 (± 8.4)	*-3.4*	*-10.7*	*-23.3*
	2	4	19.6	85.4 (± 1.0)	105.0	78.6 (± 7.5)	1.8	6.8	8.3
	3	4	14.6	73.7 (± 1.3)	88.3	80.6 (± 11.5)	4.8	-6.9	24.5
	**average ± standard deviation**		**17.4 ± 2.6**	**77.9 ± 6.4**	**95.4 ± 8.6**	**81.5 ± 3.5**	**1.0 ± 4.1**	**-3.6 ± 9.2**	**3.2 ± 24.3[f] / 5.6[g]**
	1	7	43.3	50.5 (± 0.3)	93.8	50.2 (± 0.8)	6.5	0.4	13.1
	2	7	41.7	55.1 (± 2.1)	96.8	48.5 (± 8.7)	9.8	6.7	19.0
	3	7	37.7	47.1 (± 0.8)	84.8	49.7 (± 5.5)	12.6	-2.6	25.0
	**average ± standard deviation**		**40.9 ± 2.9**	**50.9 ± 4.0**	**91.8 ± 6.3**	**49.4 ± 0.9**	**9.6 ± 3.0**	**1.5 ± 4.7**	**19.1 ± 6.0[f] / 19.1[g]**
	1	18	75.4	27.2 (± 0.1)	102.6	13.9 (± 3.2)	10.7	13.3	12.4
	2	18	79.3	21.5 (± 0.0)	100.8	14.4 (± 0.5)	6.4	7.2	7.4
	3	18	76.1	22.4 (± 0.1)	98.5	12.0 (± 0.2)	11.9	10.3	13.5
	**average ± standard deviation**		**76.9 ± 2.1**	**23.7 ± 3.1**	**100.6 ± 2.1**	**13.4 ± 1.2**	**9.7 ± 2.9**	**10.3 ± 3.1**	**11.1 ± 3.3[f] / 11.1[g]**
^{13}C-labeled PBSeT (set 1)	1	4	47.8	45.1 (± 1.8)	92.9	18.4 (± 0.7)	33.8	26.7	41.5
	2	4	45.5	46.2 (± 0.0)	91.7	19.6 (± 0.8)	35.0	26.7	43.4
	3	4	46.9	37.9 (± 0.4)	84.8	19.3 (± 1.2)	33.8	18.5	41.9
	**average ± standard deviation**		**46.7 ± 1.2**	**43.1 ± 4.5**	**89.8 ± 4.4**	**19.1 ± 0.6**	**34.2 ± 0.7**	**24.0 ± 4.7**	**42.3 ± 1.0[f] / 42.3[g]**
	1	7	61.3	38.6 (± 2.1)	99.9	5.1 (± 0.0)	33.6	33.5	35.4
	2	7	61.6	34.8 (± 3.5)	96.4	5.8 (± 1.2)	32.6	28.9	34.6
	3	7	63.8	35.1 (± 4.2)	98.9	6.0 (± 0.2)	30.2	29.1	32.1
	**average ± standard deviation**		**62.2 ± 1.4**	**36.1 ± 2.1**	**98.4 ± 1.8**	**5.6 ± 0.5**	**32.1 ± 1.7**	**30.5 ± 2.6**	**34.0 ± 1.7[f] / 34.0[g]**
	1	18	74.3	20.8 (± 1.3)	95.1	2.0 (± 0.1)	23.6	18.7	24.1
	2	18	75.1	26.9 (± 0.0)	102.0	2.9 (± 0.6)	22.0	24.0	22.7
	3	18	75.3	22.5 (± 2.0)	97.8	2.2 (± 0.1)	22.5	20.3	23.0
	**average ± standard deviation**		**74.9 ± 0.5**	**23.4 ± 3.1**	**98.3 ± 3.5**	**2.4 ± 0.5**	**22.7 ± 0.8**	**21.0 ± 2.7**	**23.3 ± 0.8[f] / 23.3[g]**
^{13}C-labeled Cellulose (set 1)	1	3	46.5	42.6 (± 2.3)	89.1	-	-	-	-
	2	3	46.9	44.5 (± 0.2)	91.4	-	-	-	-
	3	3	46.4	44.8 (± 2.4)	91.2	-	-	-	-
	**average ± standard deviation**		**46.6 ± 0.3**	**44.0 ± 1.2**	**90.6 ± 1.3**				
	1	6	53.6	39.1 (± 1.0)	92.7	-	-	-	-
	2	6	54.0	43.3 (± 0.4)	97.3	-	-	-	-
	3	6	53.3	40.6 (± 2.0)	94.0	-	-	-	-
	**average ± standard deviation**		**53.6 ± 0.3**	**41.0 ± 2.1**	**94.6 ± 2.4**				
	1	18	63.0	32.1 (± 1.9)	95.1	-	-	-	-
	2	18	65.4	42.1 (± 0.5)	107.5	-	-	-	-
	3	18	69.0	34.5 (± 0.2)	103.6	-	-	-	-
	**average ± standard deviation**		**65.8 ± 3.0**	**36.2 ± 5.3**	**102.1 ± 6.4**				

Polymer	Replicate bottle	Incubation time (days)	$^{(13)}C_{mineralized}$	$^{13}C_{nonmineralized}$	Mass balance on ^{13}C[a]	$^{(13)}C_{residual\ polyester}$[b]	$^{(13)}C_{biomass}$ (estimate 1)[c]	$^{13}C_{biomass}$ (estimate 2)[d]	Estimated CUE[e]
			(% of added polyester $^{(13)}C$)						
Nonlabeled PBAT (set 2)	1	9	22.5	-	-	74.1	3.4	-	13.1
	2	9	21.3	-	-	57.1	21.6	-	50.4
	3	9	20.3	-	-	53.0	26.7	-	56.8
	**average ± standard deviation**		**21.4 ± 1.1**			**61.4 ± 11.2**	**17.2 ± 12.2**		**40.1 ± 23.6[f] / 44.6[g]**
	1	14	50.6	-	-	30.9	18.5	-	26.8
	2	14	50.8	-	-	36.1	13.1	-	20.5
	3	14	46.7	-	-	45.3	8.1	-	14.7
	**average ± standard deviation**		**49.3 ± 2.3**			**37.4 ± 7.3**	**13.2 ± 5.2**		**20.7 ± 6.0[f] / 21.1[g]**
	1	23	82.3	-	-	4.4	13.4	-	14.0
	2	23	85.3	-	-	2.7	12.0	-	12.4
	3	23	85.5	-	-	2.6	11.9	-	12.3
	**average ± standard deviation**		**84.4 ± 1.8**			**3.2 ± 1.0**	**12.4 ± 0.8**		**12.9 ± 1.0[f] / 12.9[g]**
Nonlabeled PBSeT (set 2)	1	9	20.8	-	-	57.8	21.3	-	50.6
	2	9	21.1	-	-	56.0	22.9	-	52.0
	3	9	24.3	-	-	64.5	11.2	-	31.5
	**average ± standard deviation**		**22.1 ± 1.9**			**59.5 ± 4.5**	**18.5 ± 6.3**		**44.7 ± 11.5[f] / 45.5[g]**
	1	12	44.6	-	-	27.9	27.5	-	38.1
	2	12	48.2	-	-	30.1	21.7	-	31.0
	3	12	46.0	-	-	36.5	17.5	-	27.6
	**average ± standard deviation**		**46.3 ± 1.8**			**31.5 ± 4.4**	**22.2 ± 5.0**		**32.2 ± 5.4[f] / 32.4[g]**
	1	20	82.7	-	-	1.9	15.4	-	15.7
	2	20	87.7	-	-	0.8	11.5	-	11.6
	3	20	87.4	-	-	1.0	11.6	-	11.7
	**average ± standard deviation**		**85.9 ± 2.8**			**1.3 ± 0.6**	**12.8 ± 2.2**		**13.0 ± 2.3[f] / 13.0[g]**
Nonlabeled PHBH (set 2)	1	5	23.7	-	-	40.1	36.2	-	60.5
	2	5	25.1	-	-	57.0	17.9	-	41.6
	3	5	25.8	-	-	30.5	43.8	-	62.9
	**average ± standard deviation**		**24.9 ± 1.1**			**42.5 ± 13.4**	**32.6 ± 13.3**		**55.0 ± 11.7[f] / 56.8[g]**
	1	8	48.1	-	-	16.8	35.2	-	42.3
	2	8	50.6	-	-	19.6	29.8	-	37.1
	3	8	49.9	-	-	22.8	27.3	-	35.3
	**average ± standard deviation**		**49.5 ± 1.3**			**19.7 ± 3.0**	**30.7 ± 4.0**		**38.2 ± 3.6[f] / 38.3[g]**
	1	23	85.2	-	-	0.2	14.6	-	14.7
	2	23	86.9	-	-	0.2	13.0	-	13.0
	3	23	91.2	-	-	0.1	8.7	-	8.7
	**average ± standard deviation**		**87.7 ± 3.1**			**0.2 ± 0.0**	**12.1 ± 3.1**		**12.1 ± 3.1[f] / 12.1[g]**

[a] Mass balance on ^{13}C corresponds to the sum of $^{13}C_{mineralized}$ and $^{13}C_{nonmineralized}$ in percent of total substrate ^{13}C added and thus is only available for ^{13}C-labeled substrates in experimental set 1.

[b] $^{(13)}C_{residual\ polyester}$ for the ^{13}C-labeled polyesters (set 1) i*f*s based on two extractions per incubation bottle (average ± absolute range is presented, total extracted mass ~25% of total sample mass, see Section S9.1). $C_{residual\ polyester}$ for the nonlabeled polyesters is based on one extracted sample per incubation bottle (~25% of total sample mass extracted, see Section S9.2). Treatment averages and standard deviations are reported in bold.

[c] $^{(13)}C_{biomass}$ (estimate 1) was calculated as $100 - {}^{(13)}C_{mineralized} - {}^{(13)}C_{residual\ polyester}$. These values are reported in Figure 4 in the main manuscript and were used to calculate carbon use efficiencies (CUE). This calculation was performed for both ^{13}C-labeled and nonlabeled polyesters (assuming closed mass balances for nonlabeled polyesters in set 2).

[d] $^{13}C_{biomass}$ (estimate 2) was calculated by $^{13}C_{nonmineralized} - {}^{13}C_{residual\ polyester}$, these values are not reported in the main manuscript, as this calculation can only be performed for ^{13}C-labeled polyesters in set 1. Note that this value could not be calculated for ^{13}C-labeled cellulose, as residual cellulose cannot be extracted from compost and thus no data is available for $^{13}C_{residual\ cellulose}$.

[e] Carbon use efficiencies (CUE) were calculated by $100 \cdot {}^{(13)}C_{biomass} ({}^{(13)}C_{apparent\ biomass} + {}^{(13)}C_{mineralized})^{-1}$, where the $^{(13)}C_{biomass}$ is taken as $100 - {}^{(13)}C_{mineralized} - {}^{(13)}C_{residual\ polyester}$ (values from c, estimate 1). Note that CUE values could not be calculated for ^{13}C-labeled cellulose, as residual cellulose cannot be extracted from compost and thus no data is available for $^{13}C_{residual\ cellulose}$.

[f] Mean CUE (replicate average): CUE reported as the mean ± standard deviation of the replicate bottle CUE values.

[g] CUE from mean carbon pools: CUE calculated from the mean $^{(13)}C_{biomass}$ (estimate 1) and mean $^{(13)}C_{mineralized}$ across replicates; this value can slightly differ from the mean CUE (replicate average) and corresponds to the CUE values depicted in Figure 4 in the main text.

REFERENCES

(1) Nelson, T. F.; Baumgartner, R.; Jaggi, M.; Bernasconi, S. M.; Battagliarin, G.; Sinkel, C.; Künkel, A.; Kohler, H. P. E.; McNeill, K.; Sander, M. Biodegradation of Poly(Butylene Succinate) in Soil Laboratory Incubations Assessed by Stable Carbon Isotope Labelling. *Nature Communications* **2022**, *13*. https://doi.org/10.1038/s41467-022-33064-8.

(2) Nelson, T. F.; Baumgartner, R.; Jaggi, M.; Bernasconi, S. M.; Battagliarin, G.; Sinkel, C.; Künkel, A.; Kohler, H.-P. E.; McNeill, K.; Sander, M. Biodegradation of Synthetic Aliphatic-Aromatic Polyesters in Soils: Linking Chemical Structure to Biodegradability. *Environ. Sci. Technol.* **2025**. https://doi.org/10.1021/acs.est.5c03099.

(3) Nelson, T. F.; Remke, S. C.; Kohler, H. P. E.; McNeill, K.; Sander, M. Quantification of Synthetic Polyesters from Biodegradable Mulch Films in Soils. *Environmental Science and Technology* **2019**. https://doi.org/10.1021/acs.est.9b05863.

(4) Cerri, M.; Wille, F.; Arn, S.; Bucheli, T. D.; Widmer, F.; Werz, R.; McNeill, K.; Manfrin, A.; Sander, M. An Analytical Workflow to Quantify Biodegradable Polyesters in Soils and Its Application to Incubation Experiments. *Environmental Science & Technology* **2025**, *59*, 8108–8118. https://doi.org/10.1021/acs.est.4c10664.
